# Large-scale analysis of microRNA evolution

**DOI:** 10.1186/1471-2164-13-218

**Published:** 2012-06-06

**Authors:** José Afonso Guerra-Assunção, Anton J Enright

**Affiliations:** 1 EMBL - European Bioinformatics Institute, Wellcome Trust Genome Campus, Hinxton, Cambridge, CB10 1SD, United Kingdom; 2 PDBC, Instituto Gulbenkian de Ciência, Rua da Quinta Grande, 6, 2780-156, Oeiras, Portugal

## Abstract

**Background:**

In animals, microRNAs (miRNA) are important genetic regulators. Animal miRNAs appear to have expanded in conjunction with an escalation in complexity during early bilaterian evolution. Their small size and high-degree of similarity makes them challenging for phylogenetic approaches. Furthermore, genomic locations encoding miRNAs are not clearly defined in many species. A number of studies have looked at the evolution of individual miRNA families. However, we currently lack resources for large-scale analysis of miRNA evolution.

**Results:**

We addressed some of these issues in order to analyse the evolution of miRNAs. We perform syntenic and phylogenetic analysis for miRNAs from 80 animal species. We present synteny maps, phylogenies and functional data for miRNAs across these species. These data represent the basis of our analyses and also act as a resource for the community.

**Conclusions:**

We use these data to explore the distribution of miRNAs across phylogenetic space, characterise their birth and death, and examine functional relationships between miRNAs and other genes. These data confirm a number of previously reported findings on a larger scale and also offer novel insights into the evolution of the miRNA repertoire in animals, and it’s genomic organization.

## Background

MiRNAs are small (19-23nt) molecules that regulate mRNAs through binding to their 3’ UTR, mediated by the RNA induced silencing (RISC) complex [1]. This binding event causes translational repression [2,3] and mRNA destabilization [4]. The effect of binding is significant down-regulation of the target, which can be readily detected at both the protein and mRNA levels [5,6]. The function of miRNAs in general appears to be as a fine-tuner of gene expression [7].

The origin of small interfering RNAs appears to pre-date the emergence of eukaryotes [8]. The miRNA repertoires seem to be independent between animals and plants, being absent in fungi. Fungi possess elements of the processing machinery but not functional miRNAs [8]. Furthermore, although both animals and plants possess miRNAs, they operate through different mechanisms [9]. Expansions in morphological complexity in metazoans have previously been shown to correlate with expansions in miRNA repertoire [10]. This seems to indicate that miRNAs are particularly advantageous for defining cell and tissue types. In this study we focus exclusively on animal miRNAs. In recent years, animal miRNAs have been implicated in many areas of biology such as: tissue specificity, cell-fate, pluripotency, development, cancer, disease and stress response.

One of the first features observed for mature miRNAs was their high degree of similarity across species. Many miRNA families have identical mature sequences across a wide range of species, e.g. let-7 [11]. This high-degree of similarity can hamper phylogenetic approaches. Functional constraints surrounding the seed region (6-8nt) of the miRNA represent an important fraction of their length, which is less amenable to mutational changes. While many miRNAs are present in multiple species and are highly conserved, there are a growing number of miRNAs restricted to specific lineages.

The primary transcript of a miRNA (pri-miRNA) contains stem-loop structures that are recognised and excised by the enzyme Drosha [12], giving rise to precursor miRNAs (pre-miRNAs). Comparison of pre-miRNA sequences illustrates that they are less highly conserved and hence more amenable to phylogenetic approaches than the mature sequences alone.

The primary repository for miRNA sequence data is miRBase [13]. The information in miRBase is based on primary experimental data within specific species. A miRNA discovered in one species is likely to also be present in other closely related species, but this is not always captured by miRBase. This presents a significant challenge for phylogenetic analysis, as one requires information about the presence, absence and sequence of miRNA families in many species in order to perform evolutionary analysis. The rapid growth of next-generation sequencing has made it easier to predict miRNAs but it is clear that some predicted miRNAs do not validate experimentally and as such are flagged and removed from miRBase. Previously, a number of miRNA sequences were shown to be likely false-positives and have been removed from the database.

Different miRNAs usually belong to the same family if they share the same seed sequence (i.e. nucleotides 2–8 of the mature miRNA [14]). It is believed that these miRNAs have similar targets and thus similar cellular function although they may have very different spatial and temporal expression profiles.

Recently, we developed MapMi [15], a resource for cross-species mapping and identification of homologous miRNAs across genomes. This approach overcomes many of the issues described and provides a solid foundation from which to explore syntenic and phylogenetic relationships between miRNAs across species.

In our dataset, many miRNAs (48%) are encoded as independent non-coding transcripts while the rest (52%) are encoded within the introns of protein-coding genes. Some miRNAs exist as individual molecules encoded by a single locus while others occur in transcripts encoding multiple copies of the same miRNA or multiple transcripts at different genomic loci [16]. It has been postulated that in some cases multiple loci are required to increase copy-number of specific miRNA molecules in certain circumstances (e.g. miR-430 in early development of the Zebrafish embryo [4]).

Even with the rapid expansion of sequencing data available, we are still lacking a global overview of the genomic organization of miRNAs across a broad range of species, and an overview of their evolutionary relationships. Most previous studies (reviewed in [16]), focused on specific clusters in a small set of species.

Each miRNA is potentially capable of regulating hundreds (or even thousands) of mRNA targets simultaneously. It is therefore important that their regulation be tightly controlled. Moreover, it has been postulated that intronic miRNAs may regulate the same biological pathway as their host genes. Several examples of this have been found, namely in the regulation of Myosin expression [17] and cholesterol biosynthesis [18]. This suggests that miRNAs that are consistently co-localised with proteins might be involved in the same biological processes.

In this study, we performed for the first time, an automated, large-scale analysis of miRNA synteny and evolutionary associations. We use these data to explore both the arrangement and significance of miRNA loci throughout evolution. We also aim to identify those miRNA families, which have expanded or contracted in specific lineages. ly, we have performed phylogenetic profile analysis [19] to identify miRNA:miRNA and miRNA:protein pairs which appear to be significantly associated at a functional level.

We employ Dollo parsimony [20] to detect instances of miRNA family gains throughout evolution. Using these data we explore the genomic organization, evolution and functional associations of miRNAs. This data forms part of a larger and more detailed resource that can be accessed at www.ebi.ac.uk/enright-srv/Sintra. We will continue to update this resource, as more genomes become available.

## Results

Large-scale analysis of miRNA evolution and syntenic arrangement requires accurate information about the presence or absence of miRNA loci across many species. We addressed that by expanding the miRBase loci annotation using our MapMi approach [15]. The 80 species considered for these analyses are shown in Additional file 1: Table S1. One factor hampering analysis can arise from low-coverage genomes [21,22] which makes mapping and identification of miRNAs difficult. Even though the methods used for the analyses described herein are robust to gene loss, we look at all available genomes for completeness, specifying where results are likely due to a genome being low-coverage (Additional file 1: Table S1).

Our dataset is based on Ensembl [23] and Ensembl Metazoa [24] genomic sequences and protein family annotations (Ensembl Families). Annotations for miRNAs were obtained by mapping all metazoan sequences in miRBase [13] using MapMi [15] (see Methods). The dataset contains 52 species containing both protein coding annotation and miRNA annotation, and 28 species where just miRNA annotation is present. This corresponds to 774,002 protein coding loci and 31,237 miRNA coding loci across all species under analysis. Given that many miRNAs are present in multiple related copies it is essential that we can accurately place them into families. Hence, we have defined 3,053 miRNA families based on all miRNAs in our dataset (see Methods).

## Evolution of the microRNA repertoire

**Figure 1:**
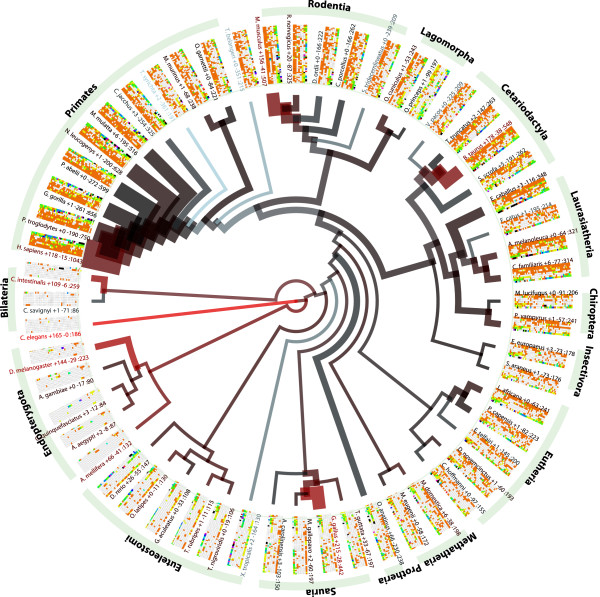
**Evolutionary Distribution of miRNA Families.** Phylogenetic tree representing miRNA family gains and losses. Branch width represents the number of miRNA families present among leaves of the branch, while the colour represents significant miRNA family loss (blue) or gain (red). For each of 408 miRNA families present at multiple loci it at least two species, we also build a graphical ‘glyph’. This glyph can be used to quickly assess presence, absence or expansion of families between clades. Each square represents a specific miRNA family. Squares are coloured as follows: white, indicates that this species does not contain a particular family, black indicates that this species contains at least 10 copies of miRNAs within that family. Copies between 1 and 10 are indicated as a rainbow gradient (red through violet). Groups of species are labelled according to the name of the evolutionary branch preceding them.

Analysis of synteny conservation (described below) provides one view of the evolution of miRNAs. We can also take a different perspective, such as assessing how miRNA genes are generated and lost across many species. This kind of analysis has been severely hampered in the past due to poor coverage of miRNAs in many species. Using our expanded dataset, we computed miRNA presence and absence profiles. These were used to perform Dollo parsimony analysis (see Methods), to infer the most likely nodes in a phylogenetic tree where miRNA families appeared (Figure 1).

One drawback of this approach is that, while we seek to detect miRNA orthologues across species, we cannot detect novel miRNAs present in species that have been poorly characterised at the miRNA level. This creates an issue for analysis of gains and losses due to these sampling biases. Some species are extremely well profiled for small RNAs while for others there exists little or no validated data. However for those sets of species which are well profiled, such analyses can still provide useful information about the evolutionary dynamics of miRNA families.

The results of this analysis are striking and show a large number of miRNA expansions across the phylogenetic tree (Figure 1). As previously reported [10], we observe a significant increase in miRNA number as morphological complexity increases with significant growth starting for metazoans and in particular across eutheria [10]. The largest growth is observed for rodents and primates with a significant gain observed for great apes (see Figure 1). Globally the tree highlights sampling biases between clades. Some clades (e.g. Mammals) are well profiled while others (e.g. Insectivora, Bilateria) are poorly profiled. Individual species (e.g. Tarsius syrichta) although they are in a well-profiled clade may have poor assemblies that hamper miRNA identification. Hence care must be taken in the interpretation of miRNA repertoire and the prediction of large gains and losses.

Additionally, we observe gains within Insects and Nematodes; this is particularly striking due to the absence of many species in these groups in the phylogenetic tree. A small number of clades exhibit significant losses, such as frog, marsupials, squirrel and hedgehog. Some of these perceived losses are most likely due to poor miRNA characterization within these species that, possibly due to assembly problems, cannot be recovered by the MapMi pipeline.

## Evolutionary comparison of miRNA genomic context

**Figure 2:**
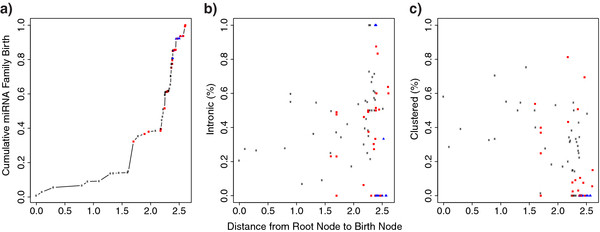
**Evolution of miRNA families.** For each node of the phylogenetic tree represented in Figure 1, a series of properties were assessed. Internal nodes are represented by black dots. Terminal nodes corresponding to high-coverage genomes are represented by red squares, while low-coverage genomes are represented by blue triangles. a) Cumulative number of miRNA families appearing at each node. b) Percentage of appearing miRNA families that are intronic per node. c) Percentage of appearing miRNA families that are part of miRNA clusters per node.

The results obtained by applying Dollo parsimony, for each miRNA family, were combined with genomic context annotations to assess how these spread out across evolution. The phylogenetic distance (branch-length) between the root node and the other nodes was taken as a proxy for node age. As previously reported [25], we observe major miRNA expansions in the bilaterian and vertebrate splits. We also observe a tendency for more recent miRNA families to be intronic rather than intergenic, whilst ancestral miRNA families tend to be found clustered more often than more recent ones (see Figure 2).

## Recently expanded miRNA families

The CAFE algorithm [26] was used to detect rapidly expanding families within specific clades (see Methods). In particular, we have focused on three clades: primates (Table 1), fish and insects (Table 2). A large number of expansions were detected in primates (Table 1) most significantly for embryonic stem (ES) cell expressed and repeat associated miRNA familes.

**Table 1:** Primate specific miRNA family expansions

**Family**	**Family members**	**Description**
SF00001	mir-1186,mir-1186b,mir-130,mir-1303,mir-130a,mir-130b,mir-130c, mir-1972,mir-301,mir-301a,mir-301b,mir-301c,mir-3090,mir-3590, mir-4452,mir-5095,mir-5096,mir-544,mir-544a,mir-544b,mir-619	ES Cell Expressed
SF00003	mir-1283,mir-1283a,mir-1283b,mir-290,mir-291a,mir-291b, mir-292,mir-293,mir-294,mir-371,mir-371b,mir-373,mir-512, mir-515,mir-516,mir-516a,mir-516b,mir-517,mir-517a,mir-517b, mir-517c,mir-518a,mir-518b,mir-518c,mir-518d,mir-518e,mir-518f, mir-519a,mir-519b,mir-519c,mir-519d,mir-519e,mir-519f,mir-520a, mir-520b,mir-520c,mir-520d,mir-520e,mir-520f,mir-520g,mir-520h, mir-521,mir-522,mir-523,mir-523a,mir-523b,mir-524, mir-525,mir-526a,mir-526b,mir-527	ES Cell Expressed Maternal Zygotic transition
SF00022	mir-1254,mir-1268,mir-1273,mir-1273c,mir-1273d,mir-1273e, mir-1273f,mir-1273g,mir-1304,mir-297,mir-297a,mir-297b,mir-297c, mir-4419b,mir-4459,mir-4478,mir-466,mir-466a,mir-466b,mir-466c, mir-466d,mir-466e,mir-466f,mir-466g,mir-466h,mir-466i,mir-466j, mir-466k,mir-466l,mir-466m,mir-466n,mir-466o,mir-466p,mir-467a,mir-467b,mir-467c,mir-467d,mir-467e,mir-467g,mir-467h,mir-566,mir-669a,mir-669b,mir-669c,mir-669d,mir-669e,mir-669f,mir-669g,mir-669h,mir-669i,mir-669j,mir-669k,mir-669l,mir-669m,mir-669o,mir-669p	Repeat Associated miRNAs (simple repeats, SINE, LTR)
SF00030	mir-2284a,mir-2284b,mir-2284c,mir-2284d,mir-2284e,mir-2284f,mir-2284g,mir-2284h,mir-2284i,mir-2284k,mir-2284l,mir-2284m,mir-2284n,mir-2284o,mir-2284p,mir-2284q,mir-2284r,mir-2284s,mir-2284t,mir-2284v,mir-2284w,mir-2284x,mir-2285a,mir-2285b,mir-2285c,mir-2285d,mir-2312,mir-2435,mir-548a,mir-548ab,mir-548ac,mir-548ad,mir-548ae,mir-548ag,mir-548ah,mir-548ai,mir-548aj,mir-548ak,mir-548al,mir-548am,mir-548an,mir-548b,mir-548c,mir-548d,mir-548e,mir-548f,mir-548g,mir-548h,mir-548i,mir-548j,mir-548k,mir-548l,mir-548m,mir-548n,mir-548o,mir-548p,mir-548q,mir-548t,mir-548u,mir-548v,mir-548w,mir-548x,mir-548y,mir-570,mir-603	Repeat Associated miRNAs (MADE1 elements)
SF00037	mir-3586	
SF00069	mir-1261,mir-1302,mir-1302b,mir-1302c,mir-1302d,mir-1302e	MER 63 Repeat Associated miRNAs
SF00090	mir-1587	Unknown
SF00099	mir-3585,mir-463,mir-465,mir-465a,mir-465b,mir-465c,mir-470, mir-506,mir-507,mir-508,mir-509,mir-509a,mir-509b,mir-510, mir-513a,mir-513b,mir-513c,mir-514,mir-514b,mir-547,mir-652, mir-742,mir-743a,mir-743b,mir-871,mir-878,mir-880,mir-881, mir-883,mir-883a,mir-883b,mir-888,mir-890,mir-892,mir-892a,mir-892b	X-linked miRNA cluster
SF00160	mir-378b,mir-378d,mir-378f,mir-378g	Unknown
SF00227	mir-4426	Unknown
SF00280	mir-703	Unknown
SF00332	mir-1233	Unknown
SF00335	mir-4310	Unknown
SF00379	mir-1244	Unknown
SF00386	mir-4646	Unknown
SF00447	mir-1236	Unknown
SF00481	mir-1973,mir-4485	Unknown
SF00485	mir-4640	Unknown
SF00731	mir-3118	Unknown
SF00807	mir-4509	Unknown
SF00912	mir-663,mir-663a,mir-663b	Tumor Suppressor
SF00954	mir-3689a,mir-3689c,mir-3689d,mir-3689e,mir-3689f	Unknown
SF01055	mir-877	miRtron, Unknown
SF01979	mir-3675	Unknown
SF01987	mir-3180	Unknown

**Table 2:** MiRNA family expansions in Amphibians, Fish and Insects

**Clade**	**Family**	**Family members**	**Description**
Amphibian	SF00050	mir-427	Maternal Zygotic Switch
Fish	SF00051	mir-430a,mir-430b,mir-430c,mir-430i	Maternal Zygotic Switch
Fish	SF01291	mir-2185	Unknown
Insects	SF01286	mir-2951	Unknown expansion in Culex

Two large families of miRNAs appear to have expanded rapidly in primates. The first cluster (Table 1) contains miR-130 and miR-301 miRNAs which have been previously reported [25] as ancient miRNAs arising from tandem repeat duplications and which have been remodeled in animals. Members of this primate expanded family have been shown to have ES cell expression [27,28]. The second cluster is also linked to ES cell expression and contains members such as miR-290 – miR-294. Interestingly, not only is the miR-290-294 set of miRNAs expressed in ES cells but it has been postulated to be a putative maternal zygotic switching mechanism in mouse oocytes [29].

It is intriguing that such families of miRNAs involved in pluripotency and early embryonic development have expanded in primates, and it mirrors expansions seen for other maternal zygotic switches described below for Insects and Fish. The increase in both morphological complexity and longevity in primates possibly requires increasingly complex control of gene-expression in stem cells. These results suggest that miRNAs are expanding in unison [30].

Aside from these two groups of ES cell related miRNAs we observe significant expansion of two large families of repeat associated miRNAs. It has previously been shown that Alu elements were expanded in the ancestor of Old and New World monkeys and that this facilitated expansion of segmental duplications [31]. Other studies have shown that such Alu expansion might also support frequent duplication of short units such as miRNAs [32].

The first cluster contains a number of miRNAs derived from simple repeats, (LINE and LTR elements), which have previously been shown to have expanded in primates, again likely through segmental duplication. The second family contains miRNAs likely derived from MADE1 elements, while the third family contains MER63 derived miRNAs [33]. These data further support the hypothesis that many primate expanded miRNA families are derived from repetitive elements and formed through rounds of segmental duplication. The relevance and function of such miRNAs is difficult to establish. One possibility that has been suggested before is that such repeats may act as generators of novel miRNA sequences which have yet to find functional relevance.

Another interesting expansion involves a family of X-linked miRNAs including miR-465 and miR-509. A large number of expansions are also listed for miRNAs whose function and expression are not well characterised yet (Tables 1 and 2). A number of other expansions are observed for other miRNA families, however in many cases little is known about the family members involved.

For fish, amphibians and insects, few expansions are detected (Table 2). However, two out of the four detected expansions involve miRNA families implicated in the Maternal-Zygotic transition, a process in early development that is regulated by miRNAs [4]. In particular miR-430 has been reported to have rapidly expanded in *Danio rerio*. We also detect a similar expansion for the equivalent MZ-switch miRNA in *Xenopus tropicalis* (miR-427). An expansion is also detected for miR-2185 in *Danio rerio*, however this miRNA has been poorly characterised with limited expression information pointing to a possible role in heart development. For insects a single expansion is detected within *Aedes* for miR-2951, however this miRNA is also poorly characterised.

## Synteny analysis

Analysis of linkage and synteny is a useful tool for establishing both orthology relationships and also functional linkages between genes. The application of synteny analysis to miRNA genes (both intronic and intergenic) has not been applied previously on a large scale. We used the Enredo [34] algorithm to segment genomes into homologous collinear regions that include both protein-coding and miRNA genes. Enredo is a graph-based system for detecting collinear segments in genome sequences that handles large-scale genome rearrangements such as duplications and deletions. Enredo does not compute the likely history of genome-rearrangements but forms a solid basis for such analyses by providing a stable set of co-linear segment blocks.

We explored the question of whether synteny blocks containing miRNAs showed differences compared to those blocks that contain solely protein-coding genes. Moreover, we wanted to assess whether particular species illustrated unexpected arrangements for miRNA genes when compared to other species.

## Syntenic blocks containing microRNAs

**Figure 3:**
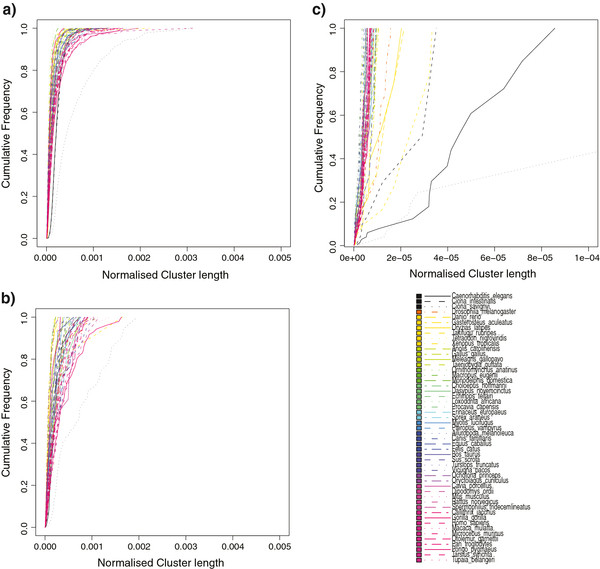
**Normalised Cluster Length per Species.** Cumulative plots of miRNA cluster size (in bp/genome size) per species, normalised by total genome length. Species are coloured according to the grouping shown in Figure 1 and listed in Additional file 1: Table S1; **(a)** clusters that contain only protein coding genes; **(b)** Mixed clusters containing both protein coding and miRNA genes; **(c)** Clusters containing only miRNA loci.

Some of the earliest analysis on genomic synteny and rearrangement was performed by Nadeau and Taylor [35] with subsequent work by Sankoff [36]. Similarly, we computed block-length distributions (Figure 3) for all genomes for three distinct classes of synteny blocks (i) Protein-coding only blocks (ii) Mixed blocks (encoding both miRNA and protein coding genes) and (iii) miRNA only blocks. For protein-coding only blocks we observe the expected distributions of block-lengths that have been previously described by Nadeau and Taylor. The majority of blocks are small, and extremely long blocks are rare, approximating a power-law distribution. Blocks that encode only miRNAs have a different distribution where long blocks occur at a higher frequency, giving a bimodal distribution where both short and long blocks are favored. Mixed blocks predominantly follow the observed patterns seen for protein-coding only blocks but again have more long blocks than expected. Genome compaction among fish is readily observable (Additional file 2: Figure S2) for both protein-coding and mixed blocks, hence we normalise (see Methods) for total genome size (Figure 3). For mixed blocks the only outlier is *Ciona savignyi*, which exhibits longer than expected blocks, however this may in fact be due to poor genome assembly. Interestingly, for miRNA-only blocks, most species exhibit similar block length distributions, except for *C. elegans, C. intestinalis, C. savignyi, D. melanogaster* and D. *rerio,* T. *rubripes* and O. *latipes*. These species have the smallest genomes in the dataset yet would seem to have longer miRNA encoding blocks than expected. This finding suggests that miRNA encoded blocks may not have been subject to genome compaction and appear to be relatively stable in terms of length across species and independent of genome size. One possibility is that miRNA syntenic blocks are already at a maximal compaction state and hence do not appear to be affected by genome compaction.

A large fraction (59%) of the miRNA loci in our dataset are found to be encoded on the genome by transcripts containing several miRNA loci. As expected, a large fraction (63%) of these are found in conserved synteny blocks across two or more species. A small fraction (3%) of non-clustered miRNA loci are found to be in conserved synteny, albeit with protein coding genes.

**Figure 4:**
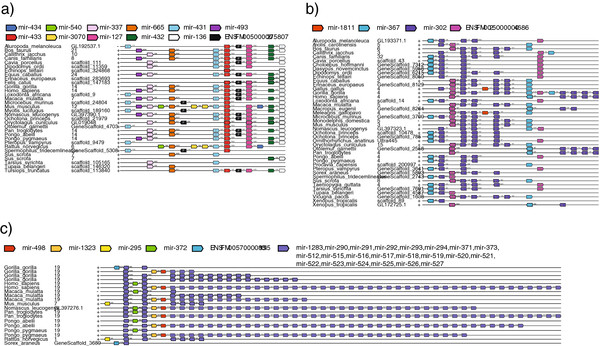
**Examples of Synteny Block Structure.** Example illustrations of the genomic organization of miRNA loci. Colours correspond to gene family. Intronic miRNA loci are indicated with the letter I inside their element, while protein coding genes are indicated by the letter P. Clusters are sorted alphabetically according to species name and the genomic coordinates of each block are indicated.

A number of example syntenic blocks are shown (Figure 4). These striking cases were chosen to illustrate the variety of the different contexts we observe within synteny blocks. In some situations new miRNA families can appear integrated in already existing, conserved syntenic clusters, albeit on a subset of species (Mouse and Rat, Figure 4a). This cluster, in particular miR-127, has previously been shown to be involved in fetal lung development [37]. In other situations, part of a cluster duplicates locally, such as miR-302 (Figure 4b). This cluster has been widely studied and is important in the definition of human embryonic stem cells [38]. In more extreme cases, a miRNA family, containing multiple miRNAs, has significantly expanded in primates and rodents (Figure 4c). These miRNAs have also been shown to be important in ES cells and are likely involved in maternal zygotic switching in animals.

We also found clusters that duplicated within the genome, but to different chromosomes (Additional file 3: Figure S1). The organization of miRNAs between species seems to be more constrained than that of the nearby protein coding genes. Due to the diversity of possible scenarios, it is challenging to accurately reconstruct the series of events that lead to the current organization of genes [39]. In general, our data is coherent with the hypothesis that miRNA genomic organization is more conserved than expected compared to both random models and protein-coding genes [40].

## Associations between microRNAs

A number of approaches have been successfully used to predict functional associations between protein-coding genes based on both their sequence and their genomic context [41-43]. We used phylogenetic profiles to apply functional association analysis to miRNAs for the first time. In the context of protein-coding genes, these approaches have usually been applied to detect possible protein-protein interactions. In our case, we sought to determine whether miRNAs from different families and different syntenic blocks had any significant and unexpected functional associations. Phylogenetic profile analysis [19] detects functional associations between genes based on their shared presence or absence across many genomes. We applied this technique to miRNA presence and absence profiles using the BayesTraits approach [44]. Surprisingly, those miRNAs within the same syntenic block, in general, do not exhibit significant functional associations. This is likely because of the extensive conservation of miRNAs, in a way that is consistent with species phylogenies. It is therefore more interesting to look at co-evolution of miRNAs in different genomic regions, as this is not affected by strong linkage between loci.

## Phylogenetic associations among miRNAs and proteins

A small number of proteins appear to exhibit significant associations with distal miRNAs (>10kb) based on phylogenetic profile analysis (Table 3).

**Table 3:** Significant Associations between protein-coding genes and miRNAs

**miRNA**	**Family**	**Protein family**	**Family**	**Description**	**Likelihood ratio**
**SF00154**	miR-876	**ENSFM00250000004087**	**IL1A**	interleukin 1 alpha	54.311
**SF00154**	miR-876	**ENSFM00250000003359**	**CD86**	antigen	54.311
**SF00154**	miR-876	**ENSFM00440000236904**	**ASGR1**	Asialoglycoprotein receptor 1	49.285
			**MGL2**	Macrophage galactose N-acetyl-galactosamine specific lectin 2	
**SF00154**	miR-876	**ENSFM00500000270948**	**MEFV**	Mediterranean fever	49.285
**SF01198**	miR-1251	**ENSFM00250000000393**	**PRAME**	Preferentially Expressed Antigen in Melanoma	53.283
**SF01004**	miR-1788	**ENSFM00500000279147**	**TLCD2**	TLC domain containing 2	49.614

The associations detected are for three independent miRNA families (miR-876, miR-1251 and miR-1788). The associations for miR-876 are particularly interesting as there are four detected and all the protein-coding genes involved play a role in immune response. Two of the proteins, IL1A and CD86 have well established roles in immune response (Cytokine signaling and T-cell receptor signaling). The ASGR1 protein appears to be involved in endocytosis of glycoproteins and is a target of the Hepatitis virus. MGL2 is a C-type lectin active in Macrophages. Finally MEFV is a protein producing Pyrin in white blood cells (eosinophils and monocytes) and appears to play a role in inflammation. Mutations in the MEFV gene cause the Mediterranean fever an inflammatory disease. While the miR-876 associations appear to have strong connections to immune response, little is known about the expression or activity of miR-876. The only experimentally validated target so far for this miRNA in human is MCL1 (Induced myeloid leukemia cell differentiation) [45], while predicted regulatory targets of this miRNA from both MicroCosm and TargetScan [13,46] indicate a preference for receptor proteins.

Similarly, the miR-1251 familiy is poorly characterised but shows an interesting association with PRAME, a protein that normally is found exclusively in testis, but that is also highly expressed in melanoma. Finally, we detected a strong association between the fish specific miRNA miR-1788 and the TLCD2 protein family. Again in this instance little is known about the miRNA and the co-evolving protein. These associations represent interesting cases for further analysis both computational and experimental.

We also searched for significant phylogenetic associations between different miRNA families. Nevertheless, after filtering of associations found based on small numbers of species, there were no significant miRNA:miRNA associations.

## Discussion

We have constructed a global synteny map and phylogenetic analysis for miRNAs across 80 animal species. The dataset used not only forms the basis of our analyses but is also, we believe, interesting and useful resource for the community. The full dataset is available at http://www.ebi.ac.uk/enright-srv/Sintra. We will continue to update this resource as new genomes and miRNAs become available and as their annotation improves.

Using these data we have undertaken a large-scale analysis of miRNA synteny, genomic organization and evolution. Our results recapitulate a number of earlier findings [25], in a fully automated fashion, with many more genomes and miRNAs. Our work revisits previous studies on the evolution of the miRNA repertoire and its correlation with morphological complexity [10], whilst also highlighting the fact that few miRNA families are shared between different clades. We show that miRNAs have atypical patterns of synteny with preferences for longer clustered regions, which do not appear to be affected by genome compaction.

We have also discovered several new features of miRNA evolution and additionally reconfirm using automated methods, the recent growth of miRNA loci in a number of animal lineages including rodents and primates and an apparent loss of miRNA families in a smaller number species such as *Xenopus tropicalis*. We find that the largest miRNA expansions detected frequently involve miRNAs involved in both pluripotency and switching from maternal to zygotic gene expression in the early embryo. Furthermore, we have performed for the first time a large-scale phylogenetic profile analysis of miRNA and proteins, discovering a number of novel associations between miRNAs and protein coding genes with implications for the roles of miRNAs in immune response. Our data also identifies quite clearly those genomes whose low-coverage or poor assembly makes them difficult to work with. Many challenges are presented by low sequence coverage of certain genomes and biases towards model species. However we believe the current results shed new light on miRNA evolution and it will be interesting to explore the effect of new genomes and better sequence assemblies over time. Additionally, further sequencing and validation of miRNA families will be useful to remove erroneously predicted miRNA families and to mitigate biases. We hope these results and our dataset will prove useful to the community.

## Materials and methods

### Dataset

We retrieved genomic sequences from all species in Ensembl [23] (version 62) and Ensembl Metazoa [24] (version 9). We used MapMi [15] (version 1.0.4) to map all the metazoan miRNAs in miRBase [13,47] (release 17) against all genomes, using the default MapMi score threshold of 35. This dataset was merged with miRBase annotations, to retain the full miRNA annotation and increase sensitivity. The protein coding data was obtained using the Ensembl API to retrieve coordinates, ID and family information for all proteins. Proteins with no family information or with ambiguous family attribution were removed from the dataset to ensure coherence of the homology attributions across species.

### Phylogenetic tree

The phylogenetic trees shown are based on the tree provided by Ensembl on http://tinyurl.com/ensembltree. This is a rooted, binary branching phylogram built from molecular data. All format conversions and node sorting necessary for compatibility with the programs used in this research were performed using the Mesquite framework for phylogenetic analysis [48].

### miRNA family attribution

To classify miRNAs in a comparable fashion, we grouped them into homologous families. All miRNA stem-loop sequences were compared using the Needleman-Wunsch algorithm (global-global alignment), as implemented in ggsearch (FASTA package) [49], using a scoring matrix that gives double weight to in-seed matching. This differentiation was performed using an expanded set of nucleotide codes in the seed region. Families are then defined by single-linkage clustering of the scores. Single-linkage clustering was chosen for its computational simplicity, and ease of interpretation of the results. The appropriate threshold was determined by minimizing the split-join distance [50] between the clustering and miRBase families. The families used in this analysis are enumerated in Additional file 4: Table S2.

### Synteny block detection and visualization

The syntenic anchor dataset was built by combining the miRNA and protein coding datasets, where each anchor is identified by its family name. The file was sorted and duplicates were eliminated according to the Enredo documentation. We detected conserved collinear segments using Enredo [34] (version 0.5) using the following options: *max-gap-length*=10000, *max-path-dissimilarity*=10, *min-regions*=2, *min-anchors*=2, simplify-graph=7. Blocks sharing a terminal anchor were chained together, according to standard operating procedures (J. Herrero, personal communication). To visualise synteny blocks, we developed a set of scripts to align the conserved synteny blocks by miRNA family using a Perl implementation of the Needleman-Wunsch algorithm producing plots using PostScript. Each anchor is coloured based on its family (e.g. see Figure 4 and Additional file 3: Figure S1).

### Association analysis

Phylogenetic profiles, as defined herein, are vectors containing, for each species, the presence or absence status per miRNA family. It has been shown [19] that gene families that are gained and lost in a correlated fashion, are often involved in the same biological processes. We studied correlated miRNA gene gains and losses by using the BayesTraits package [51] in a sequential fashion as implemented in the bms_runner script [44] (version 1.4). This approach performs a Maximum Likelihood based analysis taking into account the phylogenetic distribution of the species under analysis, removing potential biases caused by uneven sampling of the phylogenetic space.

### Birth and death of miRNA families

It is important, not only to look at the presence of miRNAs in present day species, but also to reconstruct the most likely state of the presence or absence of miRNAs in their ancestors. There are several models to infer the most parsimonious scenario [52]. The major difference between them concerns the assumptions of the model in regard to the relative birth and death rate for each gene family.

In the case of miRNA families, current data indicates a low probability of convergent evolution. Based on this, we have selected Dollo parsimony, an approach that allows each gene family to be gained once, with no restrictions on the number of times it suffers secondary loss. It is thus robust to losses due to genome assembly issues. We used this approach, as implemented in the PHYLIP package [53] (version 3.69). Binary presence/absence data for each of the miRNA families were used allowing us to obtain an estimate of the evolutionary time of birth for each of the miRNA families in our dataset. This was used to explore miRNA evolution from different perspectives, as shown in Figures 1 and 2.

### Fast expansions/deletions

While some miRNA families are present in a single copy in each genome, some families have rapidly expanded in some clades. To assess these fast expansions or unexpectedly fast deletions we use CAFE [26] (Version 2.2). This approach uses quantitative data for the number of elements of each family at each species, and requires that the gene families being studied are present at the root node of the provided phylogenetic tree. To accommodate this requirement, we performed this analysis in a selected set of sub-trees.

## Competing interests

The authors declare that they have no competing interests.

## Author’s contributions

AJE conceived the experiment. J.A.G-A performed the analyses and contributed to the design of the experiment. J.A.G-A wrote and maintains the computer programs used for the analysis. AJE and J.A.G-A wrote the manuscript and produced the figures. All authors read and approved the final manuscript.

## Supplementary Material

Additional file 1 **Table S1.**List of genomes analised in this study, including assemblyname, assembly release date, coverage depth and assembly status. This information was retrieved from the Ensembl public MySQL server. Click here for file

Additional file 2 **Figure S2.**Cluster Length per Species. As in Figure 3 but without normalisation. Click here for file

Additional file 3 **Figure S1.**Further examples of Synteny Block Structure. As in Figure 4. Click here for file

Additional file 4 **Table S2.**Table containing all miRBasemiRNA subfamilies underanalysis and their corresponding family based on our family attribution procedure (see Methods). Click here for file

## References

[B1] Kim VN MicroRNA biogenesis: coordinated cropping and dicing Nat Rev Mol Cell Biol 2005 6 376 385 15852042 10.1038/nrm1644

[B2] Lim LP Lau NC Garrett-Engele P Grimson A Schelter JM Castle J Bartel DP Linsley PS Johnson JM Microarray analysis shows that some microRNAs downregulate large numbers of target mRNAs Nature 2005 433 769 773 10.1038/nature03315 15685193

[B3] Guo H Ingolia NT Weissman JS Bartel DP Mammalian microRNAs predominantly act to decrease target mRNA levels Nature 2010 466 835 840 10.1038/nature09267 20703300 PMC2990499

[B4] Giraldez AJ Mishima Y Rihel J Grocock RJ Van Dongen S Inoue K Enright AJ Schier AF Zebrafish MiR-430 promotes deadenylation and clearance of maternal mRNAs Science 2006 312 75 79 10.1126/science.1122689 16484454

[B5] Baek D Villén J Shin C Camargo FD Gygi SP Bartel DP The impact of microRNAs on protein output Nature 2008 455 64 71 10.1038/nature07242 18668037 PMC2745094

[B6] Van Dongen S Abreu-Goodger C Enright AJ Detecting microRNA binding and siRNA off-target effects from expression data Nat Methods 2008 5 1023 1025 10.1038/nmeth.1267 18978784 PMC2635553

[B7] Kosik KS MicroRNAs and cellular phenotypy Cell 2010 143 21 26 10.1016/j.cell.2010.09.008 20887887

[B8] Shabalina SA Koonin EV Origins and evolution of eukaryotic RNA interference Trends Ecol Evol (Amst) 2008 23 578 587 10.1016/j.tree.2008.06.005 18715673 PMC2695246

[B9] Voinnet O Origin, biogenesis, and activity of plant microRNAs Cell 2009 136 669 687 10.1016/j.cell.2009.01.046 19239888

[B10] Heimberg A Sempere L Moy V Donoghue P Peterson K MicroRNAs and the advent of vertebrate morphological complexity Proceedings of the National Academy of Sciences 2008 105 2946 2950 10.1073/pnas.0712259105 PMC2268565 18287013

[B11] Pasquinelli AE Reinhart BJ Slack F Martindale MQ Kuroda MI Maller B Hayward DC Ball EE Degnan B Müller P Spring J Srinivasan A Fishman M Finnerty J Corbo J Levine M Leahy P Davidson E Ruvkun G Conservation of the sequence and temporal expression of let-7 heterochronic regulatory RNA Nature 2000 408 86 89 10.1038/35040556 11081512

[B12] Krol J Loedige I Filipowicz W The widespread regulation of microRNA biogenesis, function and decay Nat Rev Genet 2010 11 597 610 20661255 10.1038/nrg2843

[B13] Griffiths-Jones S Saini HK Van Dongen S Enright AJ miRBase: tools for microRNA genomics Nucleic Acids Res 2008 36 D154 8 10.1093/nar/gkn221 17991681 PMC2238936

[B14] Lewis BP Burge CB Bartel DP Conserved seed pairing, often flanked by adenosines, indicates that thousands of human genes are microRNA targets Cell 2005 120 15 20 10.1016/j.cell.2004.12.035 15652477

[B15] Guerra-Assunção JA Enright AJ MapMi: automated mapping of microRNA loci BMC Bioinformatics 2010 11 133 10.1186/1471-2105-11-133 20233390 PMC2858034

[B16] Olena AF Patton JG Genomic organization of microRNAs Journal of cellular physiology 2009 222 540 545 20020507 10.1002/jcp.21993 PMC4028663

[B17] van Rooij E Quiat D Johnson BA Sutherland LB Qi X Richardson JA Kelm RJ Olson EN A family of microRNAs encoded by myosin genes governs myosin expression and muscle performance Dev Cell 2009 17 662 673 10.1016/j.devcel.2009.10.013 19922871 PMC2796371

[B18] Rayner KJ Esau CC Hussain FN McDaniel AL Marshall SM van Gils JM Ray TD Sheedy FJ Goedeke L Liu X Khatsenko OG Kaimal V Lees CJ Fernández-Hernando C Fisher EA Temel RE Moore KJ Inhibition of miR-33a/b in non-human primates raises plasma HDL and lowers VLDL triglycerides Nature 2011 478 404 407 10.1038/nature10486 22012398 PMC3235584

[B19] Pellegrini M Marcotte E Thompson M Eisenberg D Yeates T Assigning protein functions by comparative genome analysis: Protein phylogenetic profiles Proc Natl Acad Sci USA 1999 96 4285 4288 10.1073/pnas.96.8.4285 10200254 PMC16324

[B20] Farris J Phylogenetic analysis under Dollo's Law Syst Biol 1977 26 77 88

[B21] Milinkovitch M Helaers R Depiereux E Tzika A Gabaldon T 2X genomes - depth does matter Genome Biol 2010 11 R16 10.1186/gb-2010-11-2-r16 20144222 PMC2872876

[B22] Vilella AJ Birney E Flicek P Herrero J Considerations for the inclusion of 2x mammalian genomes in phylogenetic analyses Genome Biol 2011 12 401 21320298 10.1186/gb-2011-12-2-401 PMC3188792

[B23] Flicek P Amode MR Barrell D Beal K Brent S Chen Y Clapham P Coates G Fairley S Fitzgerald S Gordon L Hendrix M Hourlier T Johnson N Kähäri A Keefe D Keenan S Kinsella R Kokocinski F Kulesha E Larsson P Longden I McLaren W Overduin B Pritchard B Riat HS Rios D Ritchie GRS Ruffier M Schuster M Sobral D Spudich G Tang YA Trevanion S Vandrovcova J Vilella AJ White S Wilder SP Zadissa A Zamora J Aken BL Birney E Cunningham F Dunham I Durbin R Fernández-Suarez XM Herrero J Hubbard TJP Parker A Proctor G Vogel J Searle SMJ Ensembl 2011 Nucleic Acids Res 2011 39 D800 6 10.1093/nar/gkq1064 21045057 PMC3013672

[B24] Kersey PJ Lawson D Birney E Derwent PS Haimel M Herrero J Keenan S Kerhornou A Koscielny G Kähäri A Kinsella RJ Kulesha E Maheswari U Megy K Nuhn M Proctor G Staines D Valentin F Vilella AJ Yates A Ensembl Genomes: Extending Ensembl across the taxonomic space Nucleic Acids Res 2009 38 D563 D569 19884133 10.1093/nar/gkp871 PMC2808935

[B25] Hertel J Lindemeyer M Missal K Fried C Tanzer A Flamm C Hofacker IL Stadler PF Students of Bioinformatics Computer Labs 2004 and 2005: The expansion of the metazoan microRNA repertoire BMC Genomics 2006 7 25 10.1186/1471-2164-7-25 16480513 PMC1388199

[B26] De Bie T Cristianini N Demuth JP Hahn MW CAFE: a computational tool for the study of gene family evolution Bioinformatics 2006 22 1269 1271 10.1093/bioinformatics/btl097 16543274

[B27] Houbaviy HB Murray MF Sharp PA Embryonic stem cell-specific MicroRNAs Dev Cell 2003 5 351 358 10.1016/S1534-5807(03)00227-2 12919684

[B28] Landgraf P Rusu M Sheridan R Sewer A Iovino N Aravin A Pfeffer S Rice A Kamphorst AO Landthaler M Lin C Socci ND Hermida L Fulci V Chiaretti S Foà R Schliwka J Fuchs U Novosel A Müller R-U Schermer B Bissels U Inman J Phan Q Chien M Weir DB Choksi R De Vita G Frezzetti D Trompeter H-I Hornung V Teng G Hartmann G Palkovits M Di Lauro R Wernet P Macino G Rogler CE Nagle JW Ju J Papavasiliou FN Benzing T Lichter P Tam W Brownstein MJ Bosio A Borkhardt A Russo JJ Sander C Zavolan M Tuschl T A mammalian microRNA expression atlas based on small RNA library sequencing Cell 2007 129 1401 1414 10.1016/j.cell.2007.04.040 17604727 PMC2681231

[B29] Tang F Kaneda M O'Carroll D Hajkova P Barton SC Sun YA Lee C Tarakhovsky A Lao K Surani MA Maternal microRNAs are essential for mouse zygotic development Genes Dev 2007 21 644 648 10.1101/gad.418707 17369397 PMC1820938

[B30] Roccanova L Ramphal P The role of stem cells in the evolution of longevity and its application to tissue therapy Tissue Cell 2003 35 79 81 10.1016/S0040-8166(02)00104-0 12589732

[B31] Enard W Pääbo S Comparative primate genomics Annu Rev Genomics Hum Genet 2004 5 351 378 10.1146/annurev.genom.5.061903.180040 15485353

[B32] Zhang R Wang Y-Q Su B Molecular evolution of a primate-specific microRNA family Mol Biol Evol 2008 25 1493 1502 10.1093/molbev/msn094 18417486

[B33] Yuan Z Sun X Liu H Xie J MicroRNA genes derived from repetitive elements and expanded by segmental duplication events in mammalian genomes PLoS ONE 2011 6 e17666 10.1371/journal.pone.0017666 21436881 PMC3059204

[B34] Paten B Herrero J Beal K Fitzgerald S Birney E Enredo and Pecan: genome-wide mammalian consistency-based multiple alignment with paralogs Genome Res 2008 18 1814 1828 10.1101/gr.076554.108 18849524 PMC2577869

[B35] Nadeau JH Taylor BA Lengths of chromosomal segments conserved since divergence of man and mouse Proc Natl Acad Sci USA 1984 81 814 818 10.1073/pnas.81.3.814 6583681 PMC344928

[B36] Ehrlich J Sankoff D Nadeau JH Synteny conservation and chromosome rearrangements during mammalian evolution Genetics 1997 147 289 296 9286688 10.1093/genetics/147.1.289 PMC1208112

[B37] Bhaskaran M Wang Y Zhang H Weng T Baviskar P Guo Y Gou D Liu L MicroRNA-127 modulates fetal lung development Physiological genomics 2009 37 268 278 10.1152/physiolgenomics.90268.2008 19439715 PMC2685501

[B38] Barroso-delJesus A Lucena-Aguilar G Sanchez L Ligero G Gutierrez-Aranda I Menendez P The Nodal inhibitor Lefty is negatively modulated by the microRNA miR-302 in human embryonic stem cells FASEB J 2011 25 1497 1508 10.1096/fj.10-172221 21266536

[B39] Nadeau JH Sankoff D Counting on comparative maps Trends Genet 1998 14 495 501 10.1016/S0168-9525(98)01607-2 9865155

[B40] Altuvia Y Landgraf P Lithwick G Elefant N Pfeffer S Aravin A Brownstein MJ Tuschl T Margalit H Clustering and conservation patterns of human microRNAs Nucleic Acids Res 2005 33 2697 2706 10.1093/nar/gki567 15891114 PMC1110742

[B41] Enright AJ Iliopoulos I Kyrpides NC Ouzounis CA Protein interaction maps for complete genomes based on gene fusion events Nature News 1999 402 86 90 10.1038/47056 10573422

[B42] Marcotte EM Pellegrini M Thompson MJ Yeates TO Eisenberg D A combined algorithm for genome-wide prediction of protein function Nature News 1999 402 83 86 10.1038/47048 10573421

[B43] Dandekar T Snel B Huynen M Bork P Conservation of gene order: a fingerprint of proteins that physically interact Trends in biochemical sciences 1998 23 324 328 10.1016/S0968-0004(98)01274-2 9787636

[B44] Barker D Meade A Pagel M Constrained models of evolution lead to improved prediction of functional linkage from correlated gain and loss of genes Bioinformatics 2007 23 14 20 10.1093/bioinformatics/btl558 17090580

[B45] Hsu S-D Lin F-M Wu W-Y Liang C Huang W-C Chan W-L Tsai W-T Chen G-Z Lee C-J Chiu C-M Chien C-H Wu M-C Huang C-Y Tsou A-P Huang H-D miRTarBase: a database curates experimentally validated microRNA-target interactions Nucleic Acids Res 2011 39 D163 9 10.1093/nar/gkq1107 21071411 PMC3013699

[B46] Friedman RC Farh KK-H Burge CB Bartel DP Most mammalian mRNAs are conserved targets of microRNAs Genome Res 2009 19 92 105 18955434 10.1101/gr.082701.108 PMC2612969

[B47] Kozomara A Griffiths-Jones S miRBase: integrating microRNA annotation and deep-sequencing data Nucleic Acids Res 2011 39 D152 7 10.1093/nar/gkq1027 21037258 PMC3013655

[B48] Maddison WP Maddison DR Mesquite: A modular system for evolutionary analysis Evolution 2008 62 1103 1118 10.1111/j.1558-5646.2008.00349.x 18298648

[B49] Pearson WR Lipman DJ Improved tools for biological sequence comparison Proc Natl Acad Sci USA 1988 85 2444 2448 10.1073/pnas.85.8.2444 3162770 PMC280013

[B50] Van Dongen S Graph clustering by flow simulation University of Utrecht May 2000

[B51] Barker D Pagel M Predicting functional gene links from phylogenetic-statistical analyses of whole genomes PLoS Comput Biol 2005 1 e3 10.1371/journal.pcbi.0010003 16103904 PMC1183509

[B52] Felsenstein J Parsimony in systematics: biological and statistical issues Annual review of ecology and systematics 1983 14 313 333 10.1146/annurev.es.14.110183.001525

[B53] Felsenstein J PHYLIP (phylogeny inference package), version 3.5 c Distributed by the author 1993

