# Supplementary material for: Large-scale analysis of microRNA evolution
Source: BMC Genomics. 2012 Jun 6;13:218. doi: 10.1186/1471-2164-13-218 (PMC3497579; doi:10.1186/1471-2164-13-218)
Supplement: Additional file 3 — Figure S1.Further examples of Synteny Block Structure. As in Figure 4. [file 1471-2164-13-218-S3.pdf]

	Assembly Name	Assembly Date	Coverage Depth	Assembly Mapping	Full Assembly
Acyrthosiphon_pisum	Acyr2	2008-06	high	supercontig:Acyr2|contig:Acyr2	Unassembled
Aedes_aegypti	AaegL1	2005-10	low	supercontig:AaegL1|contig:AaegL1	Unassembled
Ailuropoda_melanoleuca	ailMel1	2009-07	high	scaffold:ailMel1|contig	Unassembled
Anolis_carolinensis	AnoCar2.0	2010-05	high	scaffold:AnoCar2.0|contig:AnoCar2.0	Unassembled
Anopheles_gambiae	AgamP3	2006-02	high	scaffold:AgamP3|chunk:AgamP3	Unassembled
Apis_mellifera	Amel_2.0	2005-01	low	scaffold:Amel_2.0|contig:Amel_2.0	Unassembled
Bos_taurus	Btau_4.0	2007-10	high	chromosome:Btau_4.0|contig:Btau_4.0	Assembled
Caenorhabditis_brenneri	CB601	2007-07	high	supercontig:CB601|contig:CB601	Unassembled
Caenorhabditis_briggsae	CB3	2007-07	high	chromosome:CB3|superlink:CB3	Assembled
Caenorhabditis_elegans	WS220	2010-10	high	chromosome:WS220|clone:WS220	Assembled
Caenorhabditis_japonica	CJ302	2007-07	high	supercontig:CJ302|contig:CJ302	Unassembled
Caenorhabditis_remanei	CR2	2007-07	high	supercontig:CR2|contig:CR2	Unassembled
Callithrix_jacchus	C_jacchus3.2.1	2010-01	high	chromosome:C_jacchus3.2.1|scaffold:C_jacchus3.2.1	Assembled
Canis_familiaris	CanFam 2.0	2006-05	high	scaffold:BROADD2|contig	Unassembled
Cavia_porcellus	cavPor3	2008-03	high	scaffold:cavPor3|contig	Unassembled
Choloepus_hoffmanni	choHof1	2008-09	low	scaffold:choHof1|contig	Unassembled
Ciona_intestinalis	JGI 2	2005-03	high	chromosome:JGI2|chunk:JGI2	Assembled
Ciona_savignyi	CSAV 2.0	2005-10	high	reftig:CSAV2.0|chunk:CSAV2.0	Unassembled
Culex_quinquefasciatus	CpipJ1	2007-01	high	supercontig:CpipJ1|contig:CpipJ1	Unassembled
Danio_rerio	Zv9	2010-04	high	scaffold:Zv9|contig	Unassembled
Daphnia_pulex	Dappu1	2009-05	low	scaffold:Dappu1|contig:Dappu1	Unassembled
Dasypus_novemcinctus	dasNov2	2008-07	low	scaffold:dasNov2|contig	Unassembled
Dipodomys_ordii	dipOrd1	2008-07	low	scaffold:dipOrd1|contig	Unassembled
Drosophila_ananassae	dana_r1.3_FB2008_07	2005-08	high	scaffold:dana_r1.3_FB2008_07|chunk:dana_r1.3_FB2008_07	Unassembled
Drosophila_erecta	dere_r1.3_FB2008_07	2005-08	high	scaffold:dere_r1.3_FB2008_07|chunk:dere_r1.3_FB2008_07	Unassembled
Drosophila_grimshawi	dgri_r1.3_FB2008_07	2005-08	high	scaffold:dgri_r1.3_FB2008_07|chunk:dgri_r1.3_FB2008_07	Unassembled
Drosophila_melanogaster	BDGP 5	2006-04	high	chromosome:BDGP5|chunk:BDGP5	Assembled
Drosophila_mojavensis	dmoj_r1.3_FB2008_07	2005-08	high	scaffold:dmoj_r1.3_FB2008_07|chunk:dmoj_r1.3_FB2008_07	Unassembled
Drosophila_persimilis	dper_r1.3_FB2008_07	2005-08	high	scaffold:dper_r1.3_FB2008_07|chunk:dper_r1.3_FB2008_07	Unassembled
Drosophila_pseudoobscura	BCM-HGSC 2.8	2004-11	high	unknown_singleton:HGSC2.8|chunk:HGSC2.8	Unassembled
Drosophila_sechellia	dsec_r1.3_FB2008_07	2005-08	high	scaffold:dsec_r1.3_FB2008_07|chunk:dsec_r1.3_FB2008_07	Unassembled
Drosophila_simulans	dsim_r1.3_FB2008_07	2005-04	high	unknown:dsim_r1.3_FB2008_07|chunk:dsim_r1.3_FB2008_07	Unassembled
Drosophila_virilis	dvir_r1.2_FB2008_07	2005-08	high	scaffold:dvir_r1.2_FB2008_07|chunk:dvir_r1.2_FB2008_07	Unassembled
Drosophila_willistoni	dwil_r1.3_FB2008_07	2005-07	high	scaffold:dwil_r1.3_FB2008_07|chunk:dwil_r1.3_FB2008_07	Unassembled
Drosophila_yakuba	dyak_r1.3_FB2008_07	2005-11	high	unknown:dyak_r1.3_FB2008_07|chunk:dyak_r1.3_FB2008_07	Unassembled
Echinops_telfairi	TENREC	2005-07	low	scaffold:TENREC|contig	Unassembled
Equus_caballus	Equ Cab 2	2007-09	high	scaffold:EquCab2|contig	Unassembled
Erinaceus_europaeus	eriEur1	2006-06	low	scaffold:HEDGEHOG|contig	Unassembled
Felis_catus	CAT	2006-03	low	scaffold:CAT|contig	Unassembled
Gallus_gallus	WASHUC2	2006-05	high	chromosome:WASHUC2|contig	Assembled
Gasterosteus_aculeatus	BROAD S1	2006-02	high	group:BROADS1|contig:BROADS1	Unassembled
Gorilla_gorilla	gorGor3	2009-12	low	supercontig:gorGor3|contig	Unassembled
Homo_sapiens	GRCh37.p3	2009-02	high	chromosome:GRCh37#contig	Assembled
Ixodes_scapularis	IscaW1	2007-08	high	scaffold:IscaW1|contig:IscaW1	Unassembled
Loxodonta_africana	Loxafr3.0	2009-07	high	supercontig:loxAfr3|contig:loxAfr3	Unassembled
Macaca_mulatta	MMUL 1.0	2006-02	high	scaffold:MMUL_1|contig:MMUL_1	Unassembled
Macropus_eugenii	Meug_1.0	2008-12	low	scaffold:Meug_1.0|contig	Unassembled
Meleagris_gallopavo	Turkey_2.01	2010-09	high	scaffold:UMD2|contig:UMD2	Unassembled
Microcebus_murinus	micMur1	2007-06	low	scaffold:micMur1|contig	Unassembled
Monodelphis_domestica	monDom5	2006-10	high	scaffold:BROADO5|contig	Unassembled
Mus_musculus	NCBIM37	2007-04	high	chromosome:NCBIM37#contig	Assembled
Myotis_lucifugus	myoLuc1	2006-03	low	scaffold:MICROBAT1|contig	Unassembled
Nematostella_vectensis	Nemve1	2007-07	low	scaffold:Nemve1|contig:Nemve1	Unassembled
Nomascus_leucogenys	Nleu1.0	2010-01	high	supercontig:Nleu1.0|contig	Unassembled
Ochotona_princeps	OchPri2.0	2007-06	low	scaffold:pika|contig	Unassembled
Ornithorhynchus_anatinus	Ornithorhynchus_anatinus-5.0	2005-12	high	chromosome:OANA5|contig	Assembled
Oryctolagus_cuniculus	oryCun2	2009-11	high	scaffold:oryCun2#contig:oryCun2	Unassembled
Oryzias_latipes	HdrR	2005-10	high	chromosome:MEDAKA1|scaffold:MEDAKA1	Assembled
Otolemur_garnettii	otoGar1	2006-05	low	scaffold:BUSHBABY1|contig	Unassembled
Pan_troglodytes	CHIMP2.1	2006-03	high	chromosome:CHIMP2.1#contig	Assembled
Pediculus_humanus	PhumU1	2008-11	high	supercontig:PhumU1|contig:PhumU1	Unassembled
Pongo_abelii	PPYG2	2007-09	high	supercontig:PPYG2|contig:PPYG2	Unassembled
Pristionchus_pacificus	pp1	NA	high	supercontig:pp1|contig:pp1	Unassembled
Procavia_capensis	proCap1	2008-07	low	scaffold:proCap1|contig	Unassembled
Pteropus_vampyrus	pteVam1	2008-07	low	scaffold:pteVam1|contig	Unassembled
Rattus_norvegicus	RGSC 3.4	2004-12	high	chromosome:RGSC3.4#contig	Assembled
Saccharomyces_cerevisiae	EF 2	2010-02	high	chromosome:EF2#contig	Assembled
Schistosoma_mansoni	sma_v3.1	2008-08	low	scaffold:sma_v3.1|contig	Unassembled
Sorex_araneus	sorAra1	2005-10	low	scaffold:COMMON_SHREW1|contig	Unassembled
Spermophilus_tridecemlineatus	speTri1	2006-06	low	scaffold:SQUIRREL|contig	Unassembled
Strongylocentrotus_purpuratus	Spur2.5	2006-11	low	scaffold:Spur2.5|contig:Spur2.5	Unassembled
Sus_scrofa	Sscrofa9	2009-04	high	chromosome:Sscrofa9|contig	Assembled
Taeniopygia_guttata	Taeniopygia_guttata-3.2.4	2008-08	high	chromosome:taeGut3.2.4|contig	Assembled
Takifugu_rubripes	FUGU 4.0	2005-06	high	NA	Unassembled
Tarsius_syrichta	tarSyr1	2008-07	low	scaffold:tarSyr1|contig	Unassembled
Tetraodon_nigroviridis	TETRAODON 8.0	2007-03	high	ultracontig:TETRAODON8|contig	Unassembled
Trichoplax_adhaerens	TRIAD1	2006-08	low	scaffold:TRIAD1|contig	Unassembled
Tupaia_belangeri	tupBel1	2006-06	low	scaffold:TREESHREW|contig	Unassembled
Tursiops_truncatus	turTru1	2008-07	low	scaffold:turTru1|contig	Unassembled
Vicugna_pacos	vicPac1	2008-07	low	scaffold:vicPac1|contig	Unassembled
Xenopus_tropicalis	JGI 4.2	2009-11	high	scaffold:JGI_4.2|contig	Unassembled
