# Supplementary material for: Large-scale analysis of microRNA evolution
Source: BMC Genomics. 2012 Jun 6;13:218. doi: 10.1186/1471-2164-13-218 (PMC3497579; doi:10.1186/1471-2164-13-218)
Supplement: Additional file 4 — Table S2.Table containing all miRBasemiRNA subfamilies underanalysis and their corresponding family based on our family attribution procedure (see Methods). [file 1471-2164-13-218-S4.pdf]

miRNA Names	miR Family	miRNA Names	miR Family	miRNA Names	miR Family
bantam	SF00832	mir-1175	SF01403	mir-124b	SF00120
let-7	SF00057	mir-1178	SF00756	mir-124e	SF01656
let-7a	SF00057	mir-1179	SF01250	mir-125	SF00398
let-7b	SF00057	mir-1180	SF01632	mir-1250	SF02548
let-7c	SF00057	mir-1181	SF02555	mir-1251	SF01198
let-7d	SF00057	mir-1182	SF00995	mir-1252	SF02262
let-7e	SF00057	mir-1183	SF01680	mir-1253	SF01798
let-7f	SF00057	mir-1184	SF01406	mir-1254	SF00022
let-7g	SF00057	mir-1185	SF00031	mir-1255a	SF01494
let-7h	SF00057	mir-1186	SF00001	mir-1255b	SF00538
let-7i	SF00057	mir-1186b	SF00001	mir-1256	SF00229
let-7j	SF00057	mir-1187	SF01502	mir-1257	SF01422
let-7k	SF00057	mir-1188	SF00476	mir-1258	SF02357
lin-4	SF01193	mir-1190	SF02149	mir-125a	SF00398
lsy-6	SF01153	mir-1191	SF01509	mir-125b	SF00398
mir-1	SF00040	mir-1192	SF00919	mir-125c	SF00398
mir-10	SF00096	mir-1193	SF00031	mir-126	SF00574
mir-100	SF00096	mir-1195	SF01170	mir-1260	SF00394
mir-1000	SF00217	mir-1196	SF02245	mir-1260b	SF00583
mir-1001	SF00873	mir-1197	SF00031	mir-1261	SF00069
mir-1002	SF00853	mir-1198	SF01908	mir-1262	SF02718
mir-1003	SF01331	mir-1199	SF02442	mir-1263	SF02429
mir-1004	SF02310	mir-12	SF01761	mir-1264	SF00350
mir-1005	SF01619	mir-1200	SF02774	mir-1265	SF02095
mir-1006	SF00665	mir-1202	SF02317	mir-1266	SF02189
mir-1007	SF00734	mir-1203	SF02443	mir-1267	SF02916
mir-1008	SF00945	mir-1204	SF01584	mir-1268	SF00022
mir-1009	SF02104	mir-1205	SF01760	mir-1268b	SF01579
mir-101	SF00348	mir-1206	SF02153	mir-1269	SF01147
mir-1010	SF00196	mir-1207	SF00297	mir-1269b	SF01147
mir-1011	SF02103	mir-1208	SF01225	mir-126a	SF00574
mir-1012	SF01670	mir-122	SF00703	mir-126b	SF00574
mir-1013	SF01315	mir-1224	SF00170	mir-127	SF00420
mir-1014	SF00351	mir-1225	SF00024	mir-1270	SF00475
mir-1015	SF00231	mir-1226	SF00658	mir-1271	SF00213
mir-1016	SF02298	mir-1227	SF00270	mir-1272	SF01567
mir-1017	SF02039	mir-1228	SF00795	mir-1273	SF00022
mir-1018	SF00811	mir-1229	SF00049	mir-1273c	SF00022
mir-1019	SF00577	mir-122a	SF00703	mir-1273d	SF00022
mir-101a	SF00348	mir-122b	SF00699	mir-1273e	SF00022
mir-101b	SF00348	mir-1230	SF02004	mir-1273f	SF00022
mir-101c	SF02227	mir-1231	SF00889	mir-1273g	SF00022
mir-1020	SF02292	mir-1232	SF00640	mir-1275	SF00243
mir-1021	SF02915	mir-1233	SF00332	mir-1276	SF01336
mir-1022	SF03037	mir-1234	SF01144	mir-1277	SF00510
mir-103	SF00282	mir-1235	SF00601	mir-1278	SF02418
mir-103a	SF00282	mir-1236	SF00447	mir-1279	SF01532
mir-105	SF00101	mir-1237	SF00255	mir-128	SF00029
mir-105a	SF00101	mir-1238	SF00333	mir-1280	SF00583
mir-105b	SF00101	mir-1239	SF01135	mir-1281	SF00696
mir-106	SF00038	mir-124	SF00120	mir-1282	SF01124
mir-106a	SF00038	mir-1240	SF00261	mir-1283	SF00003
mir-106b	SF00038	mir-1241	SF00590	mir-1283a	SF00003
mir-107	SF00282	mir-1243	SF01722	mir-1283b	SF00003
mir-107a	SF00282	mir-1244	SF00379	mir-1284	SF01199
mir-107b	SF00282	mir-1245	SF01898	mir-1285	SF00635
mir-10a	SF00096	mir-1245b	SF01407	mir-1285a	SF00635
mir-10b	SF00096	mir-1246	SF00289	mir-1285b	SF00635
mir-10c	SF00096	mir-1247	SF00619	mir-1286	SF01042
mir-10d	SF00096	mir-1248	SF01183	mir-1287	SF00518
mir-11	SF01300	mir-1249	SF00449	mir-1288	SF00218
mir-1174	SF02446	mir-124a	SF00120	mir-1289	SF01203

miRNA Names	miR Family
mir-128a	SF00029
mir-128b	SF00029
mir-129	SF00448
mir-1290	SF00406
mir-1291	SF01461
mir-1291a	SF01461
mir-1291b	SF01461
mir-1292	SF00023
mir-1293	SF00793
mir-1294	SF00392
mir-1295	SF00174
mir-1296	SF00579
mir-1297	SF00543
mir-1298	SF01283
mir-1299	SF02573
mir-129a	SF00448
mir-129b	SF00448
mir-13	SF00010
mir-130	SF00001
mir-1301	SF01246
mir-1302	SF00069
mir-1302b	SF00069
mir-1302c	SF00069
mir-1302d	SF00069
mir-1302e	SF00069
mir-1303	SF00001
mir-1304	SF00022
mir-1305	SF02689
mir-1306	SF01243
mir-1307	SF00817
mir-130a	SF00001
mir-130b	SF00001
mir-130c	SF00001
mir-132	SF00498
mir-1321	SF00179
mir-1322	SF00155
mir-1323	SF00324
mir-1324	SF01029
mir-1325	SF02736
mir-1326	SF02716
mir-1327	SF02857
mir-1328	SF01370
mir-1329	SF01519
mir-133	SF00466
mir-1330	SF02377
mir-1331	SF02652
mir-1332	SF01306
mir-1333	SF02796
mir-1334	SF02380
mir-1335	SF00396
mir-1336	SF01812
mir-1337	SF01819
mir-1338	SF01758
mir-1339	SF02453
mir-133a	SF00466
mir-133b	SF00466
mir-133c	SF00466
mir-133d	SF00466
mir-134	SF00967
mir-1340	SF01095
mir-1341	SF01175
mir-1342	SF02913

miRNA Names	miR Family
mir-1343	SF00384
mir-1344	SF02382
mir-1345	SF01235
mir-1346	SF00071
mir-1347	SF02597
mir-1348	SF00788
mir-1349	SF02529
mir-135	SF00371
mir-1350	SF00996
mir-1351	SF01069
mir-1352	SF02595
mir-1353	SF00020
mir-1354	SF02933
mir-1355	SF01578
mir-1356	SF00514
mir-1357	SF00971
mir-1358	SF01263
mir-1359	SF02123
mir-135a	SF00371
mir-135b	SF00371
mir-135c	SF00371
mir-136	SF00262
mir-1360	SF02204
mir-1361	SF01435
mir-1362	SF00612
mir-1363	SF01662
mir-1364	SF03029
mir-1365	SF00515
mir-1366	SF02383
mir-1367	SF02188
mir-1368	SF02346
mir-1369	SF00066
mir-137	SF00091
mir-1370	SF02437
mir-1371	SF00410
mir-1372	SF00754
mir-1373	SF02691
mir-1374	SF01881
mir-1375	SF01799
mir-1376	SF01650
mir-1377	SF01860
mir-1378	SF02856
mir-1379	SF01265
mir-137a	SF00091
mir-137b	SF00091
mir-138	SF00070
mir-1380	SF02672
mir-1381	SF02615
mir-1382	SF01872
mir-1383	SF02216
mir-1384	SF01844
mir-1385	SF00105
mir-1386	SF00039
mir-1387	SF01684
mir-1388	SF00586
mir-1389	SF01988
mir-138a	SF00070
mir-138b	SF00070
mir-139	SF00691
mir-1390	SF01190
mir-1391	SF01975
mir-1392	SF02196

miRNA Names	miR Family
mir-1393	SF01505
mir-1394	SF01865
mir-1395	SF01664
mir-1396	SF02452
mir-1397	SF00436
mir-1398	SF02894
mir-1399	SF00126
mir-13a	SF00010
mir-13b	SF00010
mir-14	SF00032
mir-140	SF00465
mir-1400	SF02202
mir-1401	SF03049
mir-1402	SF02018
mir-1403	SF01497
mir-1404	SF02599
mir-1405	SF02288
mir-1406	SF01552
mir-1407	SF01595
mir-1408	SF00061
mir-1409	SF01945
mir-141	SF00226
mir-1410	SF02912
mir-1411	SF00718
mir-1412	SF00198
mir-1413	SF02740
mir-1414	SF00741
mir-1415	SF01484
mir-1416	SF02601
mir-1417	SF02712
mir-1418	SF02919
mir-1419a	SF00708
mir-1419b	SF00708
mir-1419c	SF00708
mir-1419d	SF00708
mir-1419e	SF00708
mir-1419f	SF00708
mir-1419g	SF00708
mir-142	SF00377
mir-1420a	SF00286
mir-1420b	SF00286
mir-1420c	SF00286
mir-1420d	SF00286
mir-1420e	SF00286
mir-1420f	SF00286
mir-1420g	SF00286
mir-1421a	SF00045
mir-1421aa	SF00045
mir-1421ab	SF00045
mir-1421ac	SF00045
mir-1421ad	SF00045
mir-1421ae	SF00045
mir-1421af	SF00045
mir-1421ag	SF00045
mir-1421ah	SF00045
mir-1421ai	SF00045
mir-1421aj	SF00045
mir-1421ak	SF00045
mir-1421al	SF00045
mir-1421am	SF00045
mir-1421b	SF00045
mir-1421c	SF00045

miRNA Names	miR Family	miRNA Names	miR Family	miRNA Names	miR Family
mir-1421d	SF00045	mir-1467	SF01830	mir-1563	SF03043
mir-1421e	SF00045	mir-1468	SF00736	mir-1564	SF02402
mir-1421f	SF00045	mir-1469	SF02618	mir-1565	SF02361
mir-1421g	SF00045	mir-146a	SF00221	mir-1566	SF00124
mir-1421h	SF00045	mir-146b	SF00221	mir-1567	SF02239
mir-1421i	SF00045	mir-146c	SF00221	mir-1568	SF02817
mir-1421j	SF00045	mir-147	SF00338	mir-1569	SF01606
mir-1421k	SF00045	mir-1470	SF01321	mir-1570	SF01706
mir-1421l	SF00045	mir-1471	SF00517	mir-1571	SF00993
mir-1421m	SF00045	mir-1473	SF02331	mir-1572	SF02099
mir-1421n	SF00045	mir-147a	SF00338	mir-1573	SF01339
mir-1421o	SF00045	mir-147b	SF00338	mir-1574	SF00172
mir-1421p	SF00045	mir-148	SF00167	mir-1575	SF02134
mir-1421q	SF00045	mir-1487	SF02372	mir-1576	SF02353
mir-1421r	SF00045	mir-148a	SF00167	mir-1577	SF02490
mir-1421s	SF00045	mir-148b	SF00167	mir-1578	SF01942
mir-1421t	SF00045	mir-149	SF00041	mir-1579	SF02425
mir-1421u	SF00045	mir-1497	SF00425	mir-1580	SF01925
mir-1421v	SF00045	mir-15	SF02635	mir-1581	SF02161
mir-1421w	SF00045	mir-150	SF01109	mir-1582	SF02560
mir-1421x	SF00045	mir-1502a	SF02327	mir-1583	SF01035
mir-1421y	SF00045	mir-1502b	SF01452	mir-1584	SF00158
mir-1421z	SF00045	mir-1502c	SF01452	mir-1585	SF01061
mir-1422a	SF00109	mir-1502d	SF01232	mir-1586	SF01589
mir-1422b	SF00109	mir-1504	SF01141	mir-1587	SF00090
mir-1422c	SF00109	mir-151	SF00006	mir-1588	SF00587
mir-1422d	SF00109	mir-151b	SF00006	mir-1589	SF01989
mir-1422e	SF00108	mir-152	SF00167	mir-1590	SF00938
mir-1422f	SF00109	mir-153	SF00337	mir-1591	SF01935
mir-1422g	SF00109	mir-1537	SF00948	mir-1592	SF01562
mir-1422h	SF00108	mir-1538	SF01515	mir-1593	SF02393
mir-1422i	SF00109	mir-1539	SF02388	mir-1594	SF01292
mir-1422j	SF00109	mir-153a	SF00337	mir-1595	SF01893
mir-1422k	SF00109	mir-153b	SF00337	mir-1596	SF00495
mir-1422l	SF00109	mir-153c	SF00337	mir-1597	SF00748
mir-1422m	SF00109	mir-154	SF00031	mir-1598	SF02454
mir-1422n	SF00109	mir-1540	SF01816	mir-1599	SF02964
mir-1422o	SF00109	mir-1541	SF01966	mir-15a	SF00084
mir-1422p	SF00109	mir-1542	SF01467	mir-15b	SF00084
mir-1422q	SF00109	mir-1543	SF00419	mir-15c	SF00084
mir-142a	SF00377	mir-1544	SF01010	mir-16	SF00150
mir-142b	SF00377	mir-1545	SF02501	mir-1600	SF01479
mir-143	SF00336	mir-1546	SF01357	mir-1601	SF02935
mir-1434	SF01262	mir-1547	SF00997	mir-1602	SF01167
mir-144	SF00672	mir-1548	SF03009	mir-1603	SF00018
mir-145	SF00178	mir-1549	SF03014	mir-1604	SF01969
mir-1451	SF01825	mir-154a	SF00031	mir-1605	SF02887
mir-1452	SF02922	mir-154b	SF00031	mir-1606	SF02275
mir-1453	SF00128	mir-155	SF00102	mir-1607	SF02148
mir-1454	SF00930	mir-1550	SF02970	mir-1608	SF02893
mir-1456	SF02042	mir-1551	SF01415	mir-1609	SF02121
mir-1457	SF01472	mir-1552	SF01057	mir-1610	SF02498
mir-1458	SF02948	mir-1553	SF00104	mir-1611	SF02208
mir-1459	SF02158	mir-1554	SF00553	mir-1612	SF00113
mir-146	SF00221	mir-1555	SF02629	mir-1613	SF02687
mir-1460	SF02566	mir-1556	SF02334	mir-1614	SF02254
mir-1461	SF01034	mir-1557	SF02859	mir-1615	SF01719
mir-1462	SF01104	mir-1558	SF00653	mir-1616	SF03026
mir-1463	SF00462	mir-1559	SF02013	mir-1617	SF02900
mir-1464	SF02517	mir-1560	SF02826	mir-1618	SF01829
mir-1465	SF00136	mir-1561	SF00715	mir-1619	SF01849
mir-1466	SF02401	mir-1562	SF00711	mir-1620	SF00692

miRNA Names	miR Family	miRNA Names	miR Family	miRNA Names	miR Family
mir-1621	SF01046	mir-1683	SF01333	mir-1741	SF01294
mir-1622	SF01807	mir-1684	SF02074	mir-1742	SF01238
mir-1623	SF00861	mir-1685	SF01012	mir-1743	SF01817
mir-1624	SF01679	mir-1686	SF02156	mir-1744	SF01737
mir-1625	SF02834	mir-1687	SF01044	mir-1745	SF01995
mir-1626	SF02240	mir-1688	SF01245	mir-1746	SF02409
mir-1627	SF02523	mir-1689	SF01748	mir-1747	SF01771
mir-1628	SF01295	mir-1690	SF00358	mir-1748	SF00078
mir-1629	SF01570	mir-1691	SF02858	mir-1749	SF01779
mir-1630	SF00480	mir-1692	SF01196	mir-1750	SF02609
mir-1631	SF01922	mir-1693	SF01050	mir-1751	SF01834
mir-1632	SF03034	mir-1694	SF00804	mir-1752	SF01430
mir-1633	SF00720	mir-1695	SF02874	mir-1753	SF02067
mir-1634	SF01648	mir-1696	SF01735	mir-1754	SF02221
mir-1635	SF00149	mir-1697	SF02051	mir-1755	SF01006
mir-1636	SF01329	mir-1698	SF01354	mir-1756a	SF01114
mir-1637	SF02646	mir-1699	SF03032	mir-1756b	SF01114
mir-1638	SF01957	mir-16a	SF00150	mir-1757	SF00378
mir-1639	SF00152	mir-16b	SF00150	mir-1758	SF02464
mir-1640	SF01820	mir-16c	SF00150	mir-1759	SF02503
mir-1641	SF00886	mir-17	SF00038	mir-1760	SF02287
mir-1642	SF00558	mir-1700	SF02551	mir-1761	SF02117
mir-1643	SF01733	mir-1701	SF02534	mir-1762	SF02082
mir-1644	SF02813	mir-1702	SF01997	mir-1763	SF01823
mir-1645	SF02313	mir-1703	SF02592	mir-1764	SF00765
mir-1646	SF01683	mir-1704	SF02634	mir-1765	SF01309
mir-1647	SF02128	mir-1705	SF01951	mir-1766	SF03041
mir-1648	SF02697	mir-1706	SF01927	mir-1767	SF00479
mir-1649	SF02145	mir-1707	SF02582	mir-1768	SF00923
mir-1650	SF02124	mir-1708	SF02488	mir-1769	SF00478
mir-1651	SF02169	mir-1709	SF02048	mir-1770	SF00907
mir-1652	SF01215	mir-1710	SF01576	mir-1771	SF01398
mir-1653	SF00834	mir-1711	SF02085	mir-1772	SF02777
mir-1654	SF02983	mir-1712	SF02788	mir-1773	SF01496
mir-1655	SF01353	mir-1713	SF02436	mir-1774	SF02664
mir-1656	SF02907	mir-1714	SF01360	mir-1775	SF03035
mir-1657	SF02336	mir-1715	SF02029	mir-1776	SF01520
mir-1658	SF02781	mir-1716	SF01634	mir-1777	SF02265
mir-1659	SF02980	mir-1717	SF01707	mir-1777a	SF00849
mir-1660	SF02743	mir-1718	SF02337	mir-1777b	SF00677
mir-1661	SF01742	mir-1719	SF02943	mir-1778	SF01861
mir-1662	SF00841	mir-1720	SF02850	mir-1779	SF01939
mir-1663	SF01688	mir-1721	SF01096	mir-1780	SF00827
mir-1664	SF01933	mir-1722	SF02990	mir-1781	SF01947
mir-1665	SF02014	mir-1723	SF02662	mir-1782	SF01911
mir-1666	SF02430	mir-1724	SF02143	mir-1783	SF01762
mir-1667	SF02237	mir-1725	SF02924	mir-1784	SF00693
mir-1668	SF01111	mir-1726	SF01853	mir-1785	SF02135
mir-1669	SF00620	mir-1727	SF02475	mir-1786	SF02502
mir-1670	SF02612	mir-1728	SF02093	mir-1787	SF02491
mir-1671	SF02396	mir-1729	SF01088	mir-1788	SF01004
mir-1672	SF02052	mir-1730	SF01636	mir-1789	SF02944
mir-1673	SF02696	mir-1731	SF01074	mir-1790	SF01290
mir-1674	SF00728	mir-1732	SF01700	mir-1791	SF01588
mir-1675	SF02535	mir-1733	SF01681	mir-1792	SF02283
mir-1676	SF00803	mir-1734	SF02190	mir-1793	SF01864
mir-1677	SF00692	mir-1735	SF02304	mir-1794	SF02833
mir-1678	SF01786	mir-1736	SF01361	mir-1795	SF01068
mir-1679	SF02318	mir-1737	SF02960	mir-1796	SF02889
mir-1680	SF01266	mir-1738	SF02276	mir-1797	SF02364
mir-1681	SF01715	mir-1739	SF02642	mir-1798	SF03006
mir-1682	SF02538	mir-1740	SF00607	mir-1799	SF02969

miRNA Names	miR Family	miRNA Names	miR Family	miRNA Names	miR Family
mir-17a	SF00038	mir-185	SF00418	mir-1943	SF02938
mir-18	SF00112	mir-186	SF01054	mir-1945	SF00119
mir-1800	SF02854	mir-187	SF01218	mir-1946a	SF01930
mir-1801	SF01189	mir-188	SF00082	mir-1946b	SF02905
mir-1802	SF00900	mir-1889	SF01626	mir-1947	SF01425
mir-1803	SF00692	mir-189	SF00222	mir-1948	SF01880
mir-1804	SF01544	mir-1890	SF00281	mir-1949	SF00916
mir-1805	SF00572	mir-1891	SF02604	mir-194a	SF00254
mir-1806	SF02669	mir-1892	SF00545	mir-194b	SF00254
mir-1807	SF02399	mir-1893	SF01674	mir-195	SF00150
mir-1808	SF02839	mir-1894	SF01668	mir-1950	SF02719
mir-1809	SF01404	mir-1895	SF02800	mir-1951	SF02748
mir-181	SF02753	mir-1896	SF01431	mir-1952	SF00563
mir-1811	SF03050	mir-1897	SF01527	mir-1953	SF02340
mir-1812	SF02819	mir-1898	SF01621	mir-1954	SF00913
mir-1813	SF00140	mir-1899	SF00215	mir-1955	SF00359
mir-1814	SF00237	mir-18a	SF00112	mir-1956	SF02201
mir-1814a	SF00122	mir-18b	SF00112	mir-1957	SF02333
mir-1814b	SF01738	mir-18c	SF00112	mir-1958	SF02481
mir-1814c	SF00211	mir-190	SF00073	mir-196	SF00064
mir-1815	SF00796	mir-1900	SF02312	mir-1960	SF00661
mir-1816	SF02033	mir-1901	SF01973	mir-1961	SF00186
mir-1817	SF01125	mir-1902	SF00187	mir-1962	SF02828
mir-1818	SF02890	mir-1903	SF02676	mir-1963	SF02378
mir-1819	SF02122	mir-1904	SF03027	mir-1964	SF02024
mir-181a	SF00374	mir-1905	SF00125	mir-1965	SF02330
mir-181b	SF00374	mir-1905a	SF00125	mir-1966	SF01454
mir-181c	SF00374	mir-1905b	SF00125	mir-1967	SF01206
mir-181d	SF00374	mir-1905c	SF00125	mir-1968	SF02820
mir-182	SF00107	mir-1906	SF01158	mir-1969	SF02908
mir-1820	SF02459	mir-1907	SF01132	mir-196a	SF00064
mir-1821	SF01961	mir-1908	SF00729	mir-196b	SF00064
mir-1822	SF01631	mir-1909	SF02414	mir-196c	SF00064
mir-1823	SF02241	mir-190a	SF00073	mir-196d	SF00064
mir-1824	SF01646	mir-190b	SF00073	mir-197	SF01001
mir-1825	SF00771	mir-191	SF00542	mir-1970	SF00893
mir-1827	SF01200	mir-1910	SF02951	mir-1971	SF00970
mir-1828	SF02663	mir-1911	SF00568	mir-1972	SF00001
mir-1829a	SF00145	mir-1912	SF01217	mir-1973	SF00481
mir-1829b	SF00145	mir-1913	SF02616	mir-1976	SF00634
mir-1829c	SF00145	mir-1914	SF00397	mir-198	SF01753
mir-183	SF00107	mir-1915	SF00244	mir-1981	SF02829
mir-1830	SF02759	mir-192	SF00434	mir-1982	SF00602
mir-1832	SF02572	mir-1923	SF03048	mir-1983	SF01916
mir-1832b	SF02571	mir-1927	SF01985	mir-199	SF00310
mir-1833	SF01036	mir-1928	SF00307	mir-1993	SF01251
mir-1834	SF01875	mir-1929	SF02069	mir-199a	SF00310
mir-1835	SF02440	mir-193	SF00162	mir-199b	SF00310
mir-1836	SF02764	mir-1930	SF00885	mir-199c	SF00310
mir-1837	SF00927	mir-1931	SF01525	mir-19a	SF00036
mir-1838	SF02088	mir-1932	SF00656	mir-19b	SF00036
mir-1839	SF00369	mir-1933	SF01914	mir-19c	SF00036
mir-184	SF00618	mir-1934	SF02186	mir-19d	SF00036
mir-1840	SF02727	mir-1935	SF00961	mir-1a	SF00040
mir-1841	SF03031	mir-1936	SF02750	mir-1b	SF00040
mir-1842	SF00242	mir-1938	SF00389	mir-1c	SF01399
mir-1843	SF00891	mir-193a	SF00162	mir-2	SF00010
mir-1843b	SF00891	mir-193b	SF00162	mir-20	SF00038
mir-1844	SF03044	mir-194	SF00254	mir-200	SF00226
mir-1845	SF00156	mir-1940	SF00088	mir-2001	SF01874
mir-184a	SF00618	mir-1941	SF01434	mir-2002	SF00405
mir-184b	SF00618	mir-1942	SF02965	mir-2003	SF01594

miRNA Names	miR Family	miRNA Names	miR Family	miRNA Names	miR Family
mir-2004	SF01244	mir-2054	SF00901	mir-2198	SF01712
mir-2005	SF01842	mir-205a	SF00791	mir-22	SF00701
mir-2006	SF00456	mir-205b	SF00791	mir-2207	SF02527
mir-2007	SF02271	mir-206	SF00040	mir-2208a	SF01059
mir-2008	SF01744	mir-207	SF00828	mir-2208b	SF01059
mir-2009	SF00881	mir-2073	SF01944	mir-2209a	SF00576
mir-200a	SF00226	mir-208	SF00342	mir-2209b	SF00576
mir-200b	SF00226	mir-208a	SF00342	mir-2209c	SF00576
mir-200c	SF00226	mir-208b	SF00342	mir-221	SF00308
mir-201	SF00503	mir-20a	SF00038	mir-2210	SF03010
mir-2010	SF02243	mir-20b	SF00038	mir-2211	SF02675
mir-2011	SF02279	mir-21	SF01015	mir-2212	SF02758
mir-2012	SF01091	mir-210	SF00598	mir-2213	SF02961
mir-2013	SF01877	mir-210b	SF02450	mir-2214	SF01269
mir-202	SF00604	mir-211	SF00416	mir-2215	SF01558
mir-2022	SF01788	mir-2110	SF02884	mir-2216	SF02955
mir-2023	SF02868	mir-2113	SF00194	mir-2217	SF02590
mir-2024a	SF00428	mir-2114	SF01727	mir-2218a	SF02807
mir-2024b	SF00428	mir-2115	SF00998	mir-2218b	SF02991
mir-2024c	SF00428	mir-2116	SF02910	mir-2219	SF02982
mir-2024e	SF00428	mir-2117	SF00139	mir-222	SF00522
mir-2024f	SF00428	mir-212	SF00472	mir-2220	SF01379
mir-2024g	SF00428	mir-2126	SF01078	mir-2221	SF02598
mir-2025	SF02415	mir-2127	SF01146	mir-2222	SF01334
mir-2026	SF02407	mir-2128	SF02458	mir-2223	SF02474
mir-2027	SF02172	mir-2129	SF02637	mir-2224	SF00431
mir-2028	SF02584	mir-2130	SF02947	mir-2225	SF02918
mir-2029	SF00738	mir-2131	SF00973	mir-2226	SF01299
mir-203	SF00326	mir-2136	SF02706	mir-2227	SF01858
mir-2030	SF02360	mir-2137	SF03047	mir-2228	SF02305
mir-2031	SF01593	mir-2139	SF01065	mir-2229	SF01098
mir-2032a	SF01892	mir-214	SF00116	mir-222a	SF00522
mir-2032b	SF01892	mir-2147b	SF02505	mir-222b	SF00522
mir-2033	SF01169	mir-215	SF00434	mir-223	SF00974
mir-2034	SF02473	mir-2159	SF01873	mir-2230	SF01854
mir-2035	SF02524	mir-216	SF00181	mir-2231	SF01693
mir-2036	SF02607	mir-2162	SF01458	mir-2232	SF00454
mir-2037	SF02673	mir-2169	SF00733	mir-2233	SF00028
mir-2038	SF01850	mir-216a	SF00181	mir-2234a	SF02568
mir-2039	SF02653	mir-216b	SF01034	mir-2234b	SF02400
mir-203a	SF00326	mir-216c	SF01034	mir-2235	SF01298
mir-203b	SF00326	mir-217	SF00315	mir-2236a	SF02545
mir-204	SF00416	mir-2176	SF02398	mir-2236b	SF02545
mir-2040a	SF00644	mir-218	SF00589	mir-2237a	SF00564
mir-2040b	SF00173	mir-2183	SF00582	mir-2237b	SF00564
mir-2041	SF00920	mir-2184	SF00239	mir-2237c	SF00564
mir-2042	SF02985	mir-2185	SF01291	mir-2238a	SF00700
mir-2043a	SF00687	mir-2186	SF00956	mir-2238b	SF00700
mir-2043b	SF00687	mir-2187	SF00034	mir-2238c	SF00700
mir-2044	SF02115	mir-2188	SF00195	mir-2238d	SF00700
mir-2045	SF01481	mir-2189	SF02496	mir-2238e	SF00700
mir-2046	SF02694	mir-218a	SF00589	mir-2239	SF02888
mir-2047	SF00301	mir-218b	SF00589	mir-224	SF00318
mir-2048	SF01894	mir-219	SF00042	mir-2240a	SF00313
mir-2049	SF01572	mir-2190	SF02745	mir-2240b	SF00313
mir-204a	SF00416	mir-2191	SF02392	mir-2240c	SF00313
mir-204b	SF00416	mir-2192	SF02537	mir-2241a	SF00313
mir-205	SF00791	mir-2193	SF00709	mir-2241b	SF00313
mir-2050	SF00268	mir-2194	SF02030	mir-2241c	SF00313
mir-2051	SF01159	mir-2195	SF02280	mir-2242	SF02489
mir-2052	SF00594	mir-2196	SF02214	mir-2243	SF00894
mir-2053	SF01960	mir-2197	SF01438	mir-2244	SF03039

miRNA Names	miR Family	miRNA Names	miR Family	miRNA Names	miR Family
mir-2245	SF03018	mir-2284x	SF00030	mir-2332	SF00636
mir-2246	SF02293	mir-2285a	SF00030	mir-2333	SF01815
mir-2247	SF00875	mir-2285b	SF00030	mir-2334	SF02832
mir-2248	SF02660	mir-2285c	SF00030	mir-2335	SF02929
mir-2249	SF02479	mir-2285d	SF00030	mir-2336	SF02699
mir-2250	SF02782	mir-2286	SF02852	mir-2337	SF01981
mir-2251	SF00313	mir-2287	SF01536	mir-2338	SF02881
mir-2252	SF02771	mir-2288	SF00717	mir-2339	SF01660
mir-2253a	SF00799	mir-2289	SF01967	mir-234	SF02066
mir-2253b	SF01665	mir-229	SF02068	mir-2340	SF02860
mir-2254	SF03038	mir-2290	SF01446	mir-2341	SF02633
mir-2255	SF02613	mir-2291	SF02805	mir-2342	SF02880
mir-2256	SF00346	mir-2292	SF03017	mir-2343	SF02080
mir-2258	SF01653	mir-2293	SF00561	mir-2344	SF02844
mir-2259	SF01627	mir-2294	SF02741	mir-2345	SF02223
mir-2260	SF02765	mir-2295	SF00646	mir-2346	SF01801
mir-2261	SF01188	mir-2296	SF02405	mir-2347	SF02034
mir-2262	SF02422	mir-2297	SF02620	mir-2348	SF02155
mir-2263	SF00313	mir-2298	SF01166	mir-2349	SF02785
mir-2264	SF01923	mir-2299	SF01642	mir-235	SF00683
mir-2265	SF02166	mir-22a	SF00701	mir-2350	SF02968
mir-2266	SF02455	mir-22b	SF00701	mir-2351	SF02285
mir-2267	SF02715	mir-23	SF00205	mir-2352	SF02139
mir-2268	SF01052	mir-230	SF01899	mir-2353	SF01659
mir-2269	SF02928	mir-2300a	SF02877	mir-2354	SF01040
mir-227	SF00950	mir-2300b	SF03052	mir-2355	SF00716
mir-2270	SF01081	mir-2301	SF02547	mir-2356	SF02460
mir-2271	SF00177	mir-2302	SF00501	mir-2357	SF02348
mir-2272	SF02344	mir-2303	SF01258	mir-2358	SF02767
mir-2273	SF01097	mir-2304	SF00190	mir-2359	SF00757
mir-2274	SF01417	mir-2305	SF00464	mir-235b	SF00683
mir-2276	SF01752	mir-2306	SF00164	mir-236	SF00404
mir-2277	SF01093	mir-2307	SF02926	mir-2360	SF02408
mir-2278	SF01501	mir-2308	SF02878	mir-2361	SF01833
mir-2279	SF01442	mir-2309	SF01754	mir-2362	SF02366
mir-228	SF01592	mir-231	SF01599	mir-2363	SF02668
mir-2280	SF00714	mir-2310	SF01565	mir-2364	SF01840
mir-2281	SF01780	mir-2311	SF02037	mir-2365	SF01390
mir-2282	SF01337	mir-2312	SF00030	mir-2366	SF02150
mir-2283	SF02703	mir-2313	SF01763	mir-2367	SF02825
mir-2284a	SF00030	mir-2314	SF02441	mir-2368	SF01628
mir-2284b	SF00030	mir-2315	SF01768	mir-2369	SF02806
mir-2284c	SF00030	mir-2316	SF02036	mir-237	SF00882
mir-2284d	SF00030	mir-2317	SF00241	mir-2370	SF00951
mir-2284e	SF00030	mir-2318	SF01764	mir-2371	SF00531
mir-2284f	SF00030	mir-2319b	SF02875	mir-2372	SF02667
mir-2284g	SF00030	mir-232	SF02339	mir-2373	SF01573
mir-2284h	SF00030	mir-2320	SF01896	mir-2374	SF00768
mir-2284i	SF00030	mir-2321	SF01257	mir-2375	SF02665
mir-2284k	SF00030	mir-2322	SF02448	mir-2376	SF01161
mir-2284l	SF00030	mir-2323	SF00595	mir-2377	SF01314
mir-2284m	SF00030	mir-2324	SF02531	mir-2378	SF00663
mir-2284n	SF00030	mir-2325a	SF00191	mir-2379	SF02521
mir-2284o	SF00030	mir-2325b	SF00797	mir-238	SF01062
mir-2284p	SF00030	mir-2325c	SF01179	mir-2380	SF01685
mir-2284q	SF00030	mir-2326	SF01905	mir-2381	SF02320
mir-2284r	SF00030	mir-2327	SF02873	mir-2382	SF02862
mir-2284s	SF00030	mir-2328	SF02270	mir-2383	SF02513
mir-2284t	SF00030	mir-2329	SF02941	mir-2384	SF01247
mir-2284u	SF01366	mir-233	SF01022	mir-2385	SF02899
mir-2284v	SF00030	mir-2330	SF01598	mir-2386	SF01934
mir-2284w	SF00030	mir-2331	SF02821	mir-2387	SF00760

miRNA Names	miR Family	miRNA Names	miR Family	miRNA Names	miR Family
mir-2388	SF01932	mir-2439	SF02974	mir-2493	SF01630
mir-2389	SF02851	mir-244	SF00353	mir-2494	SF01223
mir-239	SF02623	mir-2440	SF02307	mir-2495	SF00520
mir-2390	SF01343	mir-2441	SF03013	mir-2496	SF00621
mir-2391	SF01952	mir-2442	SF00489	mir-2497	SF02761
mir-2392	SF01781	mir-2443	SF02972	mir-2498	SF01740
mir-2393	SF00725	mir-2444	SF00355	mir-2499	SF02244
mir-2394	SF00921	mir-2445	SF00684	mir-24a	SF00222
mir-2395	SF01087	mir-2446	SF01534	mir-24b	SF00222
mir-2396	SF02512	mir-2447	SF02218	mir-25	SF00421
mir-2397	SF02165	mir-2448	SF02711	mir-250	SF01413
mir-2398	SF02574	mir-2449	SF00928	mir-2500	SF01751
mir-2399	SF02603	mir-245	SF01661	mir-2501	SF02724
mir-239a	SF00189	mir-2450a	SF00937	mir-2502	SF01569
mir-239b	SF00609	mir-2450b	SF00937	mir-2503	SF02211
mir-23a	SF00205	mir-2450c	SF00937	mir-2504	SF02090
mir-23b	SF00205	mir-2451	SF02044	mir-2505	SF01242
mir-23c	SF01906	mir-2452	SF01832	mir-2506	SF01915
mir-24	SF00222	mir-2453	SF02061	mir-2507a	SF00414
mir-240	SF00952	mir-2454	SF01092	mir-2507b	SF00983
mir-2400	SF00761	mir-2455	SF02747	mir-2508	SF01449
mir-2401	SF01273	mir-2456	SF03016	mir-2509	SF00749
mir-2402	SF02325	mir-2457	SF01557	mir-251	SF01475
mir-2403	SF00548	mir-2458	SF02914	mir-2510	SF02931
mir-2404	SF01089	mir-2459	SF03028	mir-2511	SF00695
mir-2405	SF02142	mir-246	SF02225	mir-2513a	SF01429
mir-2406	SF02717	mir-2460	SF02049	mir-2513b	SF01429
mir-2407	SF01368	mir-2461	SF03000	mir-2514	SF02040
mir-2408	SF02754	mir-2462	SF02946	mir-2515	SF00654
mir-2409	SF02866	mir-2463	SF02570	mir-2516	SF00340
mir-241	SF00085	mir-2464	SF02212	mir-2517a	SF01859
mir-2410	SF00596	mir-2465	SF02259	mir-2517b	SF01859
mir-2411	SF00899	mir-2466	SF02230	mir-2518	SF00826
mir-2412	SF01332	mir-2467	SF01654	mir-2519	SF01677
mir-2413	SF02563	mir-2468	SF02865	mir-252	SF00014
mir-2414	SF01284	mir-2469	SF03030	mir-2520	SF02802
mir-2415	SF02945	mir-247	SF01172	mir-2521	SF02688
mir-2416	SF00445	mir-2470	SF00203	mir-2522a	SF01213
mir-2417	SF02776	mir-2471	SF02803	mir-2522b	SF01213
mir-2418	SF02798	mir-2472	SF01254	mir-2523	SF02714
mir-2419	SF00884	mir-2473	SF01401	mir-2524	SF01301
mir-242	SF01671	mir-2474	SF02999	mir-2525	SF02273
mir-2420	SF00230	mir-2475	SF02958	mir-2526	SF00740
mir-2421	SF00490	mir-2476	SF00135	mir-2527	SF01550
mir-2422	SF02532	mir-2477	SF01453	mir-2528	SF01734
mir-2423	SF02057	mir-2478	SF00360	mir-2529	SF01568
mir-2424	SF00922	mir-2479	SF01607	mir-252a	SF00014
mir-2425	SF01669	mir-248	SF02194	mir-252b	SF01556
mir-2426	SF00940	mir-2480	SF02690	mir-253	SF00688
mir-2427	SF02522	mir-2481	SF02870	mir-2530	SF02641
mir-2428	SF01639	mir-2482	SF01530	mir-2531	SF00637
mir-2429	SF02222	mir-2483	SF00762	mir-2532	SF01610
mir-243	SF01041	mir-2484	SF02923	mir-2533	SF00910
mir-2430	SF02260	mir-2485	SF01470	mir-2534	SF02352
mir-2431	SF02552	mir-2486	SF02530	mir-2535	SF02059
mir-2432	SF00896	mir-2487	SF00815	mir-2535b	SF02059
mir-2433	SF00269	mir-2488	SF02779	mir-2536	SF01837
mir-2434	SF01101	mir-2489	SF02608	mir-2537	SF02463
mir-2435	SF00030	mir-249	SF01878	mir-2538	SF01831
mir-2436	SF02397	mir-2490	SF02977	mir-2539	SF01039
mir-2437	SF00685	mir-2491	SF02019	mir-254	SF02192
mir-2438	SF02238	mir-2492	SF02546	mir-2540	SF01749

miRNA Names	miR Family	miRNA Names	miR Family	miRNA Names	miR Family
mir-2541	SF01389	mir-263a	SF01013	mir-2887	SF01239
mir-2542	SF02349	mir-263b	SF00107	mir-2888	SF00059
mir-2543a	SF01302	mir-264	SF01615	mir-2889	SF01204
mir-2543b	SF01302	mir-265	SF02569	mir-289	SF00892
mir-2544	SF01392	mir-266	SF00669	mir-2890	SF01940
mir-2545a	SF01432	mir-267	SF01186	mir-2891	SF01697
mir-2545b	SF01432	mir-268	SF01476	mir-2892	SF02217
mir-2546	SF02585	mir-2681	SF01690	mir-2893	SF01790
mir-2547	SF02413	mir-2682	SF01547	mir-2894	SF02917
mir-2548	SF02315	mir-269	SF00669	mir-2895	SF02901
mir-2549	SF02102	mir-26a	SF00357	mir-2896	SF02882
mir-255	SF01184	mir-26b	SF00357	mir-2897	SF02168
mir-2550	SF01540	mir-26c	SF00357	mir-2898	SF01416
mir-2551	SF02083	mir-27	SF00079	mir-2899	SF01512
mir-2552	SF01322	mir-270	SF02297	mir-28b	SF00865
mir-2553	SF01624	mir-271	SF02579	mir-28c	SF01996
mir-2554	SF02209	mir-272	SF00585	mir-29	SF00076
mir-2555	SF01308	mir-2723	SF02164	mir-290	SF00003
mir-2556	SF02026	mir-273	SF01018	mir-2900	SF02449
mir-2557	SF02861	mir-274	SF01367	mir-2901	SF02486
mir-2558	SF01116	mir-2742	SF00622	mir-2902	SF02959
mir-2559	SF02309	mir-275	SF00460	mir-2903	SF00486
mir-256	SF01583	mir-276	SF00570	mir-2904	SF01882
mir-2560	SF01226	mir-2765	SF00752	mir-2909	SF02395
mir-2561	SF00737	mir-276a	SF00570	mir-2917	SF00106
mir-2562	SF02078	mir-276b	SF00570	mir-291a	SF00003
mir-2563	SF02381	mir-276c	SF00402	mir-291b	SF00003
mir-2564	SF01460	mir-277	SF00236	mir-292	SF00003
mir-2565	SF01673	mir-2777	SF01293	mir-293	SF00003
mir-2566a	SF01511	mir-2778a	SF00897	mir-294	SF00003
mir-2566b	SF01511	mir-2778b	SF02836	mir-2940	SF01456
mir-2567a	SF00872	mir-278	SF00844	mir-2941	SF00513
mir-2567b	SF00872	mir-2788	SF01523	mir-2942	SF00839
mir-2567c	SF00871	mir-279	SF00782	mir-2943	SF02007
mir-2568a	SF01048	mir-2790	SF00365	mir-2944	SF00840
mir-2568b	SF01048	mir-2796	SF01805	mir-2944a	SF00129
mir-2569	SF01289	mir-279a	SF00676	mir-2944b	SF00840
mir-257	SF02733	mir-279b	SF00676	mir-2945	SF01649
mir-2570	SF01726	mir-279c	SF01032	mir-2946	SF01318
mir-2571	SF02055	mir-27a	SF00079	mir-295	SF00206
mir-2572	SF02264	mir-27b	SF00079	mir-2951	SF01286
mir-2573	SF00223	mir-27c	SF00079	mir-2952	SF02426
mir-2574a	SF02587	mir-27d	SF00079	mir-2953	SF02020
mir-2574b	SF02587	mir-27e	SF00079	mir-2954	SF00253
mir-2575	SF02027	mir-28	SF00006	mir-2955	SF02011
mir-2576	SF00132	mir-280	SF01045	mir-2956	SF02369
mir-2577	SF01698	mir-281	SF00606	mir-2958	SF01839
mir-2578	SF02424	mir-282	SF01388	mir-2959	SF01970
mir-2579	SF01613	mir-283	SF01827	mir-296	SF00565
mir-258	SF02812	mir-284	SF00925	mir-2960	SF01134
mir-2580	SF00427	mir-285	SF00076	mir-2961	SF02098
mir-2581	SF00537	mir-286	SF00946	mir-2962	SF01655
mir-2582a	SF02419	mir-2861	SF01555	mir-2963	SF02723
mir-2582b	SF02906	mir-286a	SF00946	mir-2964	SF02795
mir-2583	SF00250	mir-286b	SF00946	mir-2965	SF01611
mir-2584	SF02827	mir-287	SF02219	mir-2966	SF01828
mir-259	SF01585	mir-288	SF00197	mir-2967	SF02614
mir-26	SF00357	mir-2881	SF00499	mir-2968	SF02286
mir-260	SF02925	mir-2882	SF00056	mir-2969	SF01984
mir-261	SF01928	mir-2883	SF00131	mir-297	SF00022
mir-262	SF02515	mir-2885	SF01554	mir-2970	SF02485
mir-263	SF01013	mir-2886	SF00127	mir-2971	SF02060

miRNA Names	miR Family	miRNA Names	miR Family	miRNA Names	miR Family
mir-2972	SF02248	mir-3028	SF02476	mir-3078	SF02818
mir-2973	SF02760	mir-3029	SF00837	mir-3079	SF02365
mir-2974	SF00549	mir-302a	SF00144	mir-307b	SF01948
mir-2975	SF02593	mir-302b	SF00144	mir-308	SF01437
mir-2976	SF01121	mir-302c	SF00144	mir-3080	SF02451
mir-2977	SF01507	mir-302d	SF00144	mir-3081	SF02234
mir-2978	SF02482	mir-302e	SF00444	mir-3082	SF00816
mir-2979	SF01730	mir-302f	SF00422	mir-3083	SF03051
mir-297a	SF00022	mir-303	SF03033	mir-3084	SF01868
mir-297b	SF00022	mir-3030	SF01741	mir-3085	SF00163
mir-297c	SF00022	mir-3031	SF02362	mir-3086	SF01784
mir-298	SF00869	mir-3032	SF01580	mir-3087	SF02203
mir-2980	SF02713	mir-3033	SF00597	mir-3088	SF01714
mir-2981	SF00327	mir-3034	SF02132	mir-3089	SF00755
mir-2982	SF00312	mir-3035	SF01176	mir-309	SF00148
mir-2983	SF02076	mir-3036	SF00012	mir-3090	SF00001
mir-2984	SF02466	mir-3037	SF01824	mir-3091	SF01641
mir-2985	SF00399	mir-3038	SF02045	mir-3092	SF00911
mir-2986	SF02558	mir-3039	SF00947	mir-3093	SF02993
mir-2987	SF02640	mir-304	SF00856	mir-3094	SF01439
mir-2988	SF02268	mir-3040	SF02347	mir-3095	SF01907
mir-2989	SF02063	mir-3041	SF00933	mir-3096	SF00292
mir-299	SF00031	mir-3042	SF00964	mir-3096b	SF00292
mir-2991	SF00277	mir-3043	SF01436	mir-3097	SF02125
mir-2992	SF02987	mir-3044	SF00033	mir-3098	SF02685
mir-2993	SF01491	mir-3045a	SF02773	mir-3099	SF02097
mir-2994	SF02658	mir-3045b	SF02773	mir-309a	SF00148
mir-2995	SF02277	mir-3046	SF02433	mir-309b	SF00148
mir-2996	SF02246	mir-3047	SF01216	mir-30a	SF00081
mir-2997	SF01564	mir-3048	SF02108	mir-30b	SF00081
mir-29a	SF00076	mir-3049	SF01457	mir-30c	SF00081
mir-29b	SF00076	mir-305	SF00188	mir-30d	SF00081
mir-29c	SF00076	mir-3050	SF00352	mir-30e	SF00081
mir-29d	SF00076	mir-3051	SF02229	mir-30f	SF00081
mir-29e	SF00076	mir-3052	SF02130	mir-31	SF00719
mir-2a	SF00010	mir-3053	SF02693	mir-310	SF01575
mir-2b	SF00010	mir-3054	SF02228	mir-3100	SF02814
mir-2c	SF00010	mir-3055	SF02385	mir-3101	SF01312
mir-2d	SF00043	mir-3056	SF00986	mir-3102	SF01380
mir-3	SF00148	mir-3057	SF00322	mir-3103	SF02494
mir-300	SF00031	mir-3058	SF02830	mir-3104	SF02514
mir-301	SF00001	mir-3059	SF00220	mir-3105	SF01424
mir-3015a	SF00774	mir-306	SF00847	mir-3106	SF00829
mir-3015b	SF00670	mir-3060	SF01618	mir-3108	SF01806
mir-3015c	SF00670	mir-3061	SF02151	mir-3109	SF01330
mir-3016	SF00836	mir-3062	SF00299	mir-310a	SF01575
mir-3017a	SF02062	mir-3063	SF01084	mir-310b	SF01575
mir-3017b	SF02062	mir-3064	SF01386	mir-311	SF00344
mir-3018	SF02576	mir-3065	SF00664	mir-3110	SF02940
mir-3019	SF02730	mir-3066	SF03007	mir-3112	SF02461
mir-301a	SF00001	mir-3067	SF00650	mir-3113	SF00532
mir-301b	SF00001	mir-3068	SF01026	mir-3115	SF01024
mir-301c	SF00001	mir-3069	SF02404	mir-3116	SF01209
mir-302	SF00144	mir-307	SF00298	mir-3117	SF01311
mir-3020	SF02823	mir-3070a	SF00497	mir-3118	SF00731
mir-3021	SF00556	mir-3070b	SF00497	mir-3119	SF02863
mir-3022	SF02311	mir-3072	SF02302	mir-311a	SF00344
mir-3023	SF01759	mir-3073	SF01897	mir-311b	SF00343
mir-3024	SF01408	mir-3074	SF00222	mir-311c	SF00344
mir-3025	SF01900	mir-3075	SF01982	mir-312	SF00343
mir-3026	SF01160	mir-3076	SF03045	mir-3120	SF00116
mir-3027	SF00146	mir-3077	SF02611	mir-3121	SF00403

miRNA Names	miR Family	miRNA Names	miR Family	miRNA Names	miR Family
mir-3122	SF01818	mir-3175	SF00831	mir-34	SF00007
mir-3123	SF02296	mir-3176	SF02589	mir-340	SF00902
mir-3124	SF00467	mir-3177	SF02257	mir-341	SF01028
mir-3125	SF00955	mir-3178	SF01633	mir-342	SF00305
mir-3126	SF01910	mir-3179	SF01038	mir-3422	SF00855
mir-3127	SF00645	mir-318	SF00148	mir-343	SF00395
mir-3128	SF02005	mir-3180	SF01987	mir-3431	SF02274
mir-3129	SF00183	mir-3181	SF00328	mir-3432	SF02119
mir-313	SF00343	mir-3182	SF00863	mir-344	SF00288
mir-3130	SF02808	mir-3183	SF02902	mir-344a	SF00288
mir-3131	SF00775	mir-3185	SF01720	mir-344b	SF00288
mir-3132	SF01264	mir-3186	SF00166	mir-344c	SF00288
mir-3133	SF01791	mir-3187	SF01769	mir-344d	SF00288
mir-3134	SF01347	mir-3188	SF01745	mir-344e	SF00288
mir-3135	SF02170	mir-3189	SF00953	mir-344f	SF00288
mir-3135b	SF01122	mir-3191	SF01157	mir-344g	SF00288
mir-3136	SF01219	mir-3192	SF02299	mir-345	SF00207
mir-3137	SF01275	mir-3193	SF01326	mir-346	SF00540
mir-3138	SF00883	mir-3194	SF01704	mir-347	SF02323
mir-3139	SF00864	mir-3195	SF01374	mir-3470a	SF02236
mir-314	SF00838	mir-3196	SF00362	mir-3470b	SF02518
mir-3140	SF00978	mir-3197	SF00980	mir-3471	SF01689
mir-3141	SF00316	mir-3198	SF01387	mir-3472	SF02686
mir-3142	SF01709	mir-3199	SF01136	mir-3473	SF00097
mir-3143	SF02793	mir-31a	SF00246	mir-3473b	SF02510
mir-3144	SF01992	mir-31b	SF00246	mir-3473c	SF02182
mir-3145	SF02224	mir-32	SF00208	mir-3473d	SF01482
mir-3146	SF01307	mir-320	SF00058	mir-3474	SF02300
mir-3147	SF02477	mir-3200	SF01119	mir-3475	SF00430
mir-3148	SF02942	mir-3201	SF00905	mir-3477	SF00182
mir-3149	SF00898	mir-3202	SF01601	mir-3482	SF02191
mir-315	SF01604	mir-320a	SF00058	mir-3483	SF01082
mir-3150a	SF00655	mir-320b	SF00058	mir-3485	SF01462
mir-3150b	SF00655	mir-320c	SF00058	mir-3486	SF00786
mir-3151	SF00459	mir-320d	SF00058	mir-3488	SF00546
mir-3152	SF02389	mir-320e	SF00058	mir-349	SF00450
mir-3153	SF02146	mir-322	SF00732	mir-3490	SF00552
mir-3154	SF01260	mir-323	SF00031	mir-3492	SF00842
mir-3155	SF00713	mir-3236	SF01395	mir-3499	SF00842
mir-3155b	SF00264	mir-323b	SF00031	mir-34a	SF00007
mir-3156	SF01212	mir-323c	SF00031	mir-34b	SF00007
mir-3157	SF01115	mir-324	SF00138	mir-34c	SF00007
mir-3158	SF01148	mir-325	SF00429	mir-35	SF00147
mir-3159	SF01862	mir-326	SF00016	mir-350	SF00580
mir-315a	SF01604	mir-327	SF03003	mir-3503	SF02328
mir-315b	SF02079	mir-328	SF00387	mir-351	SF01810
mir-316	SF00989	mir-328a	SF00387	mir-3523	SF02976
mir-3160	SF00876	mir-328b	SF01500	mir-3524	SF01310
mir-3161	SF02636	mir-329	SF00031	mir-3526	SF01220
mir-3162	SF00279	mir-329a	SF00031	mir-3527	SF01814
mir-3163	SF02580	mir-329b	SF00031	mir-3528	SF02176
mir-3164	SF01279	mir-33	SF00153	mir-353	SF01099
mir-3165	SF01623	mir-330	SF00184	mir-3530	SF03012
mir-3166	SF01017	mir-331	SF00705	mir-3531	SF02207
mir-3167	SF01031	mir-335	SF00458	mir-3532	SF01349
mir-3168	SF00962	mir-336	SF00252	mir-3533	SF00544
mir-3169	SF01267	mir-337	SF01785	mir-3534	SF02541
mir-317	SF01127	mir-338	SF00664	mir-3535	SF00303
mir-3170	SF02116	mir-339	SF00528	mir-3536	SF02235
mir-3171	SF01775	mir-339b	SF02992	mir-3537	SF02127
mir-3173	SF02358	mir-33a	SF00153	mir-3538	SF00833
mir-3174	SF02891	mir-33b	SF00153	mir-3539	SF03025

miRNA Names	miR Family	miRNA Names	miR Family	miRNA Names	miR Family
mir-354	SF02499	mir-3610	SF00169	mir-3685	SF01546
mir-3540	SF00370	mir-3611	SF02872	mir-3686	SF01773
mir-3541	SF01133	mir-3612	SF00680	mir-3687	SF02137
mir-3542	SF02187	mir-3613	SF01746	mir-3688	SF00704
mir-3544	SF01919	mir-3614	SF01129	mir-3689a	SF00954
mir-3545	SF00326	mir-3615	SF02179	mir-3689b	SF00924
mir-3546	SF00342	mir-3616	SF01652	mir-3689c	SF00954
mir-3547	SF01448	mir-3617	SF01027	mir-3689d	SF00954
mir-3548	SF00781	mir-3618	SF01464	mir-3689e	SF00954
mir-355	SF01094	mir-3619	SF01561	mir-3689f	SF00954
mir-3550	SF00934	mir-362	SF00082	mir-369	SF00031
mir-3551	SF00787	mir-3620	SF00048	mir-3690	SF01011
mir-3552	SF01197	mir-3621	SF01426	mir-3691	SF00880
mir-3555	SF02375	mir-3622a	SF02656	mir-3692	SF00225
mir-3558	SF01126	mir-3622b	SF02656	mir-37	SF00147
mir-3559	SF02849	mir-363	SF01075	mir-370	SF00251
mir-356	SF01378	mir-3641	SF02766	mir-371	SF00003
mir-3561	SF01980	mir-3642	SF01731	mir-3713	SF02975
mir-3562	SF02864	mir-3643	SF01539	mir-3714	SF01879
mir-3564	SF01883	mir-3644	SF01549	mir-3715	SF00245
mir-3566	SF00541	mir-3645	SF01185	mir-3716a	SF02770
mir-3568	SF00944	mir-3646	SF01743	mir-3716b	SF01797
mir-3569	SF02468	mir-3647	SF00675	mir-3717	SF02702
mir-357	SF02258	mir-3648	SF00625	mir-3718a	SF01151
mir-3572	SF01793	mir-3649	SF01976	mir-3719	SF00452
mir-3573	SF02799	mir-365	SF00285	mir-371b	SF00003
mir-3575	SF01616	mir-3650	SF01846	mir-372	SF00137
mir-3577	SF01171	mir-3651	SF01352	mir-3722	SF00413
mir-3578	SF00626	mir-3652	SF00477	mir-3726	SF01852
mir-3579	SF02092	mir-3653	SF00453	mir-3727	SF02178
mir-358	SF01876	mir-3654	SF00698	mir-3728	SF01647
mir-3580	SF00175	mir-3655	SF02930	mir-3729	SF02266
mir-3583	SF02783	mir-3656	SF01514	mir-373	SF00003
mir-3584	SF00364	mir-3657	SF02106	mir-3730	SF03046
mir-3585	SF00099	mir-3658	SF00904	mir-3733	SF02390
mir-3586	SF00037	mir-3659	SF01750	mir-3734	SF00311
mir-359	SF00483	mir-3660	SF02536	mir-3736	SF00942
mir-3590	SF00001	mir-3661	SF01418	mir-3737	SF02904
mir-3591	SF00703	mir-3662	SF01488	mir-3738	SF01274
mir-3593	SF00851	mir-3663	SF01319	mir-3739	SF02427
mir-3594	SF02316	mir-3664	SF02705	mir-374	SF01382
mir-3596	SF01359	mir-3665	SF00999	mir-3741	SF02263
mir-3596c	SF02294	mir-3666	SF01682	mir-3742	SF01574
mir-3597	SF00267	mir-3667	SF01965	mir-3743	SF02423
mir-3598	SF02162	mir-3668	SF02661	mir-3745	SF02622
mir-3599	SF02617	mir-3669	SF00072	mir-3746	SF01369
mir-35a	SF00147	mir-367	SF00426	mir-3747a	SF02757
mir-35b	SF00147	mir-3670	SF02131	mir-3747b	SF02470
mir-35c	SF00147	mir-3671	SF01694	mir-3748	SF02110
mir-35d	SF00147	mir-3672	SF02205	mir-3749	SF02370
mir-35e	SF00147	mir-3673	SF00536	mir-374a	SF01382
mir-35f	SF00147	mir-3674	SF01105	mir-374b	SF01382
mir-35g	SF00147	mir-3675	SF01979	mir-375	SF00320
mir-36	SF00147	mir-3676	SF02973	mir-3750	SF01443
mir-360	SF01106	mir-3677	SF02173	mir-3751	SF00931
mir-3601	SF00309	mir-3678	SF00083	mir-3752	SF03002
mir-3604	SF02171	mir-3679	SF01889	mir-3754	SF02762
mir-3605	SF00086	mir-3680	SF02303	mir-3764	SF02416
mir-3606	SF00005	mir-3681	SF00550	mir-3766	SF02737
mir-3607	SF01107	mir-3682	SF02242	mir-3767	SF01803
mir-3609	SF00373	mir-3683	SF02755	mir-3768	SF01808
mir-361	SF01020	mir-3684	SF02903	mir-376a	SF00031

miRNA Names	miR Family	miRNA Names	miR Family	miRNA Names	miR Family
mir-376b	SF00031	mir-3888	SF02506	mir-3974	SF03020
mir-376c	SF00031	mir-3897	SF00103	mir-3975	SF01138
mir-376d	SF00031	mir-39	SF00147	mir-3976	SF01060
mir-376e	SF00031	mir-3901	SF02549	mir-3977	SF02588
mir-377	SF00031	mir-3904	SF01493	mir-3978	SF01600
mir-3770	SF02368	mir-3906	SF02072	mir-4	SF00857
mir-3771	SF02016	mir-3907	SF01236	mir-40	SF00147
mir-3773	SF01695	mir-3908	SF01651	mir-4000a	SF00860
mir-3775	SF01077	mir-3909	SF00060	mir-4000b	SF00068
mir-3776	SF01473	mir-3910	SF02355	mir-4000c	SF00067
mir-3779	SF02557	mir-3911	SF00629	mir-4000d	SF00068
mir-378	SF00519	mir-3912	SF02465	mir-4000e	SF00075
mir-3780	SF02842	mir-3913	SF00329	mir-4000f	SF01230
mir-3781	SF01076	mir-3914	SF01341	mir-4000g	SF02003
mir-3782	SF01931	mir-3915	SF02511	mir-4000h	SF00512
mir-3783	SF02681	mir-3916	SF00228	mir-4000i	SF01480
mir-3784	SF02886	mir-3917	SF00053	mir-4001a	SF00599
mir-3785	SF02553	mir-3918	SF02950	mir-4001b	SF01590
mir-3786	SF01835	mir-3919	SF02650	mir-4001c	SF01867
mir-3787	SF01596	mir-392	SF02639	mir-4001d	SF02210
mir-3788	SF00994	mir-3920	SF02896	mir-4001e	SF00917
mir-3789	SF01030	mir-3921	SF01433	mir-4001f	SF02006
mir-378b	SF00160	mir-3922	SF02157	mir-4001g	SF02478
mir-378c	SF00519	mir-3923	SF02643	mir-4001h	SF00599
mir-378d	SF00160	mir-3924	SF02126	mir-4001i	SF01112
mir-378e	SF01551	mir-3925	SF02420	mir-4002	SF00746
mir-378f	SF00160	mir-3926	SF01344	mir-4003a	SF01155
mir-378g	SF00160	mir-3927	SF02053	mir-4003b	SF01155
mir-378h	SF01421	mir-3928	SF02291	mir-4003c	SF01156
mir-378i	SF01455	mir-3929	SF01955	mir-4003d	SF01155
mir-379	SF00031	mir-3931	SF02610	mir-4004	SF02627
mir-3790	SF01409	mir-3934	SF00263	mir-4005a	SF00614
mir-3792	SF02772	mir-3935	SF02484	mir-4005b	SF00614
mir-3794	SF02025	mir-3936	SF02578	mir-4005c	SF00614
mir-3795	SF02768	mir-3937	SF01391	mir-4006a	SF00506
mir-3796	SF03023	mir-3938	SF01645	mir-4006b	SF00063
mir-3797	SF02655	mir-3939	SF01776	mir-4006c	SF00062
mir-3798	SF02811	mir-3940	SF01796	mir-4006d	SF00506
mir-3799	SF02278	mir-3941	SF00935	mir-4006e	SF00468
mir-38	SF00147	mir-3942	SF01954	mir-4006f	SF00506
mir-380	SF00031	mir-3943	SF00991	mir-4006g	SF00535
mir-3800	SF02979	mir-3944	SF02031	mir-4008a	SF01373
mir-3801	SF00383	mir-3945	SF02160	mir-4008b	SF01373
mir-3802	SF02492	mir-3955	SF01248	mir-4008c	SF01373
mir-381	SF00031	mir-3956	SF00943	mir-4009a	SF01397
mir-3811c	SF01486	mir-3957	SF02250	mir-4009b	SF02628
mir-3817	SF00778	mir-3958	SF00031	mir-4009c	SF00823
mir-382	SF00031	mir-3959	SF00031	mir-4010	SF02282
mir-3828	SF00200	mir-3960	SF00908	mir-4011a	SF02438
mir-383	SF00649	mir-3961	SF01459	mir-4011b	SF02439
mir-3837	SF01102	mir-3962	SF02105	mir-4012	SF01016
mir-384	SF00457	mir-3963	SF00360	mir-4013a	SF02256
mir-3842	SF02562	mir-3964	SF01110	mir-4013b	SF02256
mir-3852	SF02112	mir-3965	SF02493	mir-4014	SF01211
mir-3854	SF00985	mir-3966	SF02744	mir-4015	SF01974
mir-3856	SF01150	mir-3967	SF02971	mir-4016	SF00975
mir-3859	SF02199	mir-3968	SF02520	mir-4017	SF00668
mir-3861	SF01234	mir-3969	SF01787	mir-4018b	SF02526
mir-3868	SF00643	mir-3970	SF02267	mir-4019	SF01699
mir-3873	SF01924	mir-3971	SF02154	mir-4020a	SF00526
mir-3876	SF03011	mir-3972	SF02994	mir-4020b	SF00527
mir-3885	SF00052	mir-3973	SF02136	mir-4021	SF01508

miRNA Names	miR Family	miRNA Names	miR Family	miRNA Names	miR Family
mir-4022	SF00423	mir-4086	SF00932	mir-4145	SF01131
mir-4024	SF02742	mir-4087	SF02871	mir-4146	SF01067
mir-4025	SF01384	mir-4088	SF02295	mir-4147	SF02957
mir-4026	SF01522	mir-4089	SF02751	mir-4148	SF01383
mir-4027	SF02556	mir-409	SF00031	mir-4149	SF00982
mir-4028	SF01950	mir-4090	SF02997	mir-4150	SF01993
mir-4029	SF00800	mir-4091	SF02403	mir-4151	SF01717
mir-4030	SF00100	mir-4092	SF02152	mir-4152	SF02114
mir-4031	SF02649	mir-4093	SF02996	mir-4153	SF03019
mir-4033	SF02247	mir-4094	SF02594	mir-4154	SF01597
mir-4034	SF02495	mir-4095	SF02129	mir-4155	SF02435
mir-4035	SF02937	mir-4097	SF02843	mir-4156	SF02065
mir-4036	SF00439	mir-4098	SF01822	mir-4157	SF02379
mir-4037	SF01885	mir-4099	SF02784	mir-4158	SF00437
mir-4038	SF01423	mir-409a	SF00031	mir-4159	SF02284
mir-4039	SF01402	mir-41	SF00147	mir-4160	SF02544
mir-4040	SF02746	mir-410	SF00031	mir-4162	SF01450
mir-4042	SF00611	mir-4100	SF01845	mir-4163	SF01617
mir-4043	SF01410	mir-4101	SF01800	mir-4164	SF00686
mir-4044	SF02539	mir-4103	SF01495	mir-4165	SF01777
mir-4045	SF01447	mir-4104	SF01962	mir-4166	SF01721
mir-4046	SF02329	mir-4105	SF00259	mir-4168	SF03024
mir-4047	SF02032	mir-4106	SF01214	mir-4169	SF02504
mir-4048	SF02932	mir-4108	SF00293	mir-4171	SF02012
mir-4049	SF01317	mir-4109	SF02232	mir-4172	SF01903
mir-4050	SF02631	mir-411	SF00031	mir-4173	SF01605
mir-4051	SF02838	mir-4110	SF01571	mir-4174	SF01921
mir-4052	SF00401	mir-4111	SF00569	mir-4176	SF01324
mir-4053	SF01377	mir-4112	SF02261	mir-4177	SF00011
mir-4054	SF01736	mir-4113	SF01723	mir-4178a	SF02752
mir-4055	SF01272	mir-4114	SF02949	mir-4178b	SF02752
mir-4056	SF02198	mir-4115	SF01725	mir-4179	SF00848
mir-4057	SF01870	mir-4116	SF00906	mir-4180	SF02434
mir-4058	SF02670	mir-4117	SF00247	mir-4181	SF02952
mir-4059	SF01841	mir-4118	SF02081	mir-4182	SF01066
mir-4060	SF02869	mir-411b	SF00031	mir-4183	SF02674
mir-4061	SF03022	mir-412	SF00031	mir-4184	SF00123
mir-4062	SF02519	mir-4120	SF00743	mir-4185	SF01304
mir-4063	SF02709	mir-4121	SF01772	mir-4186	SF02692
mir-4064	SF03040	mir-4122	SF00764	mir-4187	SF02883
mir-4065	SF01325	mir-4123	SF01537	mir-4189	SF01477
mir-4066	SF02725	mir-4124	SF02507	mir-4190	SF01365
mir-4067	SF02775	mir-4125	SF02921	mir-4191	SF02174
mir-4068	SF01517	mir-4126	SF02778	mir-4192	SF00753
mir-4069	SF03036	mir-4127	SF01728	mir-4193	SF02421
mir-4070	SF01856	mir-4128	SF02892	mir-4194	SF00098
mir-4071	SF02022	mir-4129	SF02120	mir-4195	SF02835
mir-4072	SF01528	mir-4130	SF02575	mir-4196	SF02824
mir-4073	SF02542	mir-4131	SF02624	mir-4197	SF02677
mir-4074	SF00812	mir-4132	SF00879	mir-4198	SF00415
mir-4075	SF02586	mir-4133	SF01991	mir-4199	SF02810
mir-4076	SF01451	mir-4134	SF03008	mir-41b	SF00147
mir-4077a	SF00388	mir-4135	SF02909	mir-42	SF00147
mir-4077b	SF00388	mir-4136	SF02412	mir-4201	SF00273
mir-4077c	SF00388	mir-4137	SF02885	mir-4202	SF01261
mir-4077d	SF00388	mir-4138	SF01708	mir-4203	SF01348
mir-4078	SF02141	mir-4139	SF01936	mir-4204	SF02600
mir-4079	SF00435	mir-4140	SF02185	mir-4205	SF01794
mir-4081	SF02528	mir-4141	SF01705	mir-4206	SF01521
mir-4083	SF01918	mir-4142	SF00133	mir-4207	SF02096
mir-4084	SF02701	mir-4143	SF00745	mir-4208	SF01328
mir-4085	SF02554	mir-4144	SF02897	mir-4209	SF02816

miRNA Names	miR Family	miRNA Names	miR Family	miRNA Names	miR Family
mir-421	SF00350	mir-4294	SF00494	mir-4422	SF00356
mir-4211	SF01609	mir-4295	SF00411	mir-4423	SF01358
mir-4212	SF00432	mir-4296	SF00547	mir-4424	SF02954
mir-4213	SF00724	mir-4297	SF00114	mir-4425	SF02107
mir-4214	SF01071	mir-4298	SF02953	mir-4426	SF00227
mir-4215	SF01718	mir-4299	SF01541	mir-4427	SF01445
mir-4216	SF02678	mir-429b	SF00226	mir-4428	SF01692
mir-4217	SF02978	mir-42a	SF00496	mir-4429	SF01767
mir-4218	SF01869	mir-42b	SF01381	mir-4430	SF02432
mir-4219	SF01766	mir-43	SF00055	mir-4431	SF02175
mir-4220	SF01612	mir-4300	SF01978	mir-4432	SF01994
mir-422a	SF00482	mir-4301	SF01533	mir-4433	SF03001
mir-423	SF00651	mir-4302	SF00093	mir-4434	SF00487
mir-423a	SF00651	mir-4303	SF00110	mir-4435	SF01281
mir-424	SF00732	mir-4304	SF02301	mir-4436a	SF01103
mir-425	SF00726	mir-4305	SF01070	mir-4436b	SF01103
mir-4251	SF00859	mir-4306	SF00843	mir-4437	SF00710
mir-4252	SF01770	mir-4307	SF01162	mir-4438	SF01350
mir-4253	SF00272	mir-4308	SF01021	mir-4439	SF02801
mir-4254	SF01890	mir-4309	SF00707	mir-4440	SF02472
mir-4255	SF00319	mir-430a	SF00051	mir-4441	SF01941
mir-4256	SF00121	mir-430b	SF00051	mir-4442	SF00025
mir-4257	SF00077	mir-430c	SF00051	mir-4443	SF00488
mir-4258	SF00185	mir-430i	SF00051	mir-4444	SF00960
mir-4259	SF01543	mir-431	SF00814	mir-4445	SF02797
mir-4260	SF00027	mir-4310	SF00335	mir-4446	SF01297
mir-4261	SF00135	mir-4311	SF01139	mir-4447	SF00304
mir-4262	SF01713	mir-4312	SF02722	mir-4448	SF00219
mir-4263	SF00825	mir-4313	SF00571	mir-4449	SF01757
mir-4264	SF00168	mir-4314	SF00180	mir-4450	SF01123
mir-4265	SF01128	mir-4315	SF02708	mir-4451	SF00080
mir-4266	SF00087	mir-4316	SF00557	mir-4452	SF00001
mir-4267	SF00521	mir-4317	SF00491	mir-4453	SF01303
mir-4268	SF02290	mir-4318	SF00780	mir-4454	SF00199
mir-4269	SF02831	mir-4319	SF00287	mir-4455	SF00117
mir-427	SF00050	mir-432	SF00390	mir-4456	SF00354
mir-4270	SF01716	mir-4320	SF01747	mir-4457	SF02837
mir-4271	SF00141	mir-4321	SF02376	mir-4458	SF01058
mir-4272	SF01227	mir-4322	SF01553	mir-4459	SF00022
mir-4273	SF01510	mir-4323	SF00209	mir-4460	SF00968
mir-4274	SF01559	mir-4324	SF02101	mir-4461	SF00382
mir-4275	SF00505	mir-4325	SF01516	mir-4462	SF01165
mir-4276	SF00870	mir-4326	SF01696	mir-4463	SF00529
mir-4277	SF01338	mir-4327	SF00813	mir-4464	SF01222
mir-4278	SF02682	mir-4328	SF00468	mir-4465	SF02193
mir-4279	SF00331	mir-4329	SF00647	mir-4466	SF00616
mir-428	SF00095	mir-433	SF00502	mir-4467	SF01342
mir-4280	SF01804	mir-4330	SF02540	mir-4468	SF00523
mir-4281	SF00151	mir-4331	SF00210	mir-4469	SF01710
mir-4282	SF01687	mir-4333	SF01591	mir-4470	SF01756
mir-4283	SF01137	mir-4334	SF02023	mir-4471	SF02780
mir-4284	SF01471	mir-4335	SF02483	mir-4472	SF00015
mir-4285	SF02002	mir-4336	SF01340	mir-4473	SF02058
mir-4286	SF01474	mir-4337	SF02428	mir-4474	SF01411
mir-4287	SF00662	mir-434	SF02720	mir-4475	SF02322
mir-4288	SF00306	mir-44	SF00216	mir-4476	SF02967
mir-4289	SF01195	mir-4417	SF00463	mir-4477a	SF01191
mir-429	SF00226	mir-4418	SF00089	mir-4477b	SF01191
mir-4290	SF01253	mir-4419a	SF00330	mir-4478	SF00022
mir-4291	SF00240	mir-4419b	SF00022	mir-4479	SF01009
mir-4292	SF01231	mir-4420	SF02252	mir-448	SF00671
mir-4293	SF00224	mir-4421	SF00516	mir-4480	SF02739

miRNA Names	miR Family	miRNA Names	miR Family	miRNA Names	miR Family
mir-4481	SF00539	mir-453	SF00031	mir-465c	SF00099
mir-4482	SF00660	mir-4530	SF02371	mir-466	SF00022
mir-4483	SF00391	mir-4531	SF00363	mir-4660	SF01838
mir-4484	SF01774	mir-4532	SF02417	mir-4661	SF01958
mir-4485	SF00481	mir-4533	SF02986	mir-4662a	SF01983
mir-4486	SF01037	mir-4534	SF00026	mir-4663	SF01419
mir-4487	SF00271	mir-4535	SF00161	mir-4664	SF02017
mir-4488	SF01240	mir-4536	SF01802	mir-4665	SF00772
mir-4489	SF02804	mir-4537	SF01629	mir-4666	SF01968
mir-449	SF00008	mir-4538	SF01629	mir-4667	SF00509
mir-4490	SF00966	mir-4539	SF01782	mir-4668	SF02462
mir-4491	SF02732	mir-454	SF00232	mir-4669	SF01686
mir-4492	SF02728	mir-4540	SF01938	mir-466a	SF00022
mir-4493	SF01108	mir-454a	SF00232	mir-466b	SF00022
mir-4494	SF01943	mir-454b	SF00232	mir-466c	SF00022
mir-4495	SF02363	mir-455	SF00712	mir-466d	SF00022
mir-4496	SF00890	mir-455b	SF00712	mir-466e	SF00022
mir-4497	SF00742	mir-456	SF01351	mir-466f	SF00022
mir-4498	SF00835	mir-457a	SF00779	mir-466g	SF00022
mir-4499	SF02822	mir-457b	SF00150	mir-466h	SF00022
mir-449a	SF00008	mir-458	SF00094	mir-466i	SF00022
mir-449b	SF00008	mir-459	SF00238	mir-466j	SF00022
mir-449c	SF00008	mir-46	SF00606	mir-466k	SF00022
mir-449d	SF01005	mir-460	SF01217	mir-466l	SF00022
mir-45	SF00216	mir-4606	SF02845	mir-466m	SF00022
mir-450	SF00642	mir-460a	SF01217	mir-466n	SF00022
mir-4500	SF00433	mir-460b	SF01217	mir-466o	SF00022
mir-4501	SF01327	mir-461	SF00895	mir-466p	SF00022
mir-4502	SF01821	mir-462	SF01278	mir-466q	SF00446
mir-4503	SF02497	mir-463	SF00099	mir-4670	SF00613
mir-4504	SF02841	mir-4632	SF01711	mir-4671	SF01643
mir-4505	SF00809	mir-4633	SF00638	mir-4672	SF00559
mir-4506	SF00822	mir-4634	SF00624	mir-4673	SF02035
mir-4507	SF00159	mir-4635	SF00915	mir-4674	SF00909
mir-4508	SF02815	mir-4636	SF01271	mir-4675	SF00283
mir-4509	SF00807	mir-4637	SF02367	mir-4676	SF01241
mir-450a	SF00642	mir-4638	SF01811	mir-4677	SF00830
mir-450b	SF00642	mir-4639	SF02010	mir-4678	SF03053
mir-450c	SF00642	mir-4640	SF00485	mir-4679	SF01990
mir-451	SF00300	mir-4641	SF00639	mir-467a	SF00022
mir-4510	SF02091	mir-4642	SF01371	mir-467b	SF00022
mir-4511	SF02721	mir-4643	SF00929	mir-467c	SF00022
mir-4512	SF00914	mir-4644	SF00248	mir-467d	SF00022
mir-4513	SF01252	mir-4645	SF00926	mir-467e	SF00022
mir-4514	SF00551	mir-4646	SF00386	mir-467f	SF00202
mir-4515	SF00959	mir-4647	SF00939	mir-467g	SF00022
mir-4516	SF02043	mir-4648	SF02289	mir-467h	SF00022
mir-4517	SF01201	mir-4649	SF01739	mir-468	SF01587
mir-4518	SF02789	mir-465	SF00099	mir-4680	SF00744
mir-4519	SF00380	mir-4650	SF02350	mir-4681	SF02936
mir-451a	SF00642	mir-4651	SF00409	mir-4682	SF00977
mir-452	SF00874	mir-4652	SF02680	mir-4683	SF01851
mir-4520b	SF02853	mir-4653	SF01007	mir-4684	SF01427
mir-4521	SF02335	mir-4654	SF01412	mir-4685	SF00321
mir-4522	SF01977	mir-4655	SF01638	mir-4686	SF01051
mir-4523	SF00862	mir-4656	SF01120	mir-4687	SF00990
mir-4524	SF00630	mir-4657	SF00819	mir-4688	SF01701
mir-4525	SF02683	mir-4658	SF02769	mir-4689	SF01895
mir-4526	SF01208	mir-4659a	SF01142	mir-4690	SF02001
mir-4527	SF03042	mir-4659b	SF01142	mir-4691	SF01149
mir-4528	SF02314	mir-465a	SF00099	mir-4692	SF00443
mir-4529	SF00375	mir-465b	SF00099	mir-4693	SF01025

miRNA Names	miR Family	miRNA Names	miR Family	miRNA Names	miR Family
mir-4694	SF01194	mir-4754	SF02341	mir-4815	SF00776
mir-4695	SF00806	mir-4755	SF01513	mir-4816	SF02183
mir-4696	SF02086	mir-4756	SF01033	mir-4825	SF01080
mir-4697	SF01778	mir-4757	SF01313	mir-483	SF01466
mir-4698	SF00628	mir-4758	SF01143	mir-484	SF00941
mir-4699	SF02386	mir-4759	SF02215	mir-4847	SF02846
mir-47	SF00606	mir-4760	SF00735	mir-4848a	SF02054
mir-470	SF00099	mir-4761	SF02564	mir-4848b	SF02306
mir-4700	SF01489	mir-4762	SF02879	mir-4849	SF01789
mir-4701	SF00193	mir-4763	SF00690	mir-485	SF00031
mir-4703	SF01008	mir-4764	SF01691	mir-4850	SF01920
mir-4704	SF01836	mir-4765	SF00673	mir-4851	SF02567
mir-4705	SF03021	mir-4766	SF00074	mir-4852	SF02734
mir-4706	SF02543	mir-4767	SF00017	mir-4853	SF00275
mir-4707	SF01277	mir-4768	SF01224	mir-4854	SF02255
mir-4708	SF01202	mir-4769	SF02133	mir-486	SF01444
mir-4709	SF00588	mir-4770	SF00766	mir-4863	SF02911
mir-471	SF01003	mir-4771	SF02077	mir-487a	SF00031
mir-4710	SF01296	mir-4772	SF00852	mir-487b	SF00031
mir-4711	SF02373	mir-4773	SF00877	mir-488	SF00689
mir-4712	SF01233	mir-4774	SF01485	mir-489	SF00759
mir-4713	SF02073	mir-4775	SF00578	mir-49	SF00722
mir-4714	SF00903	mir-4776	SF02206	mir-490	SF00204
mir-4715	SF00266	mir-4777	SF02981	mir-4908	SF01356
mir-4716	SF01014	mir-4778	SF01428	mir-4909	SF01956
mir-4717	SF02487	mir-4779	SF02249	mir-491	SF00492
mir-4718	SF00784	mir-4780	SF01056	mir-4910	SF02666
mir-4719	SF01964	mir-4781	SF02410	mir-4911	SF03005
mir-4720	SF00727	mir-4782	SF01468	mir-4912	SF00936
mir-4721	SF01524	mir-4783	SF00633	mir-4913	SF02989
mir-4722	SF01622	mir-4784	SF01288	mir-4914	SF02927
mir-4723	SF00846	mir-4785	SF01581	mir-4915	SF02630
mir-4724	SF01640	mir-4786	SF00965	mir-4916	SF01917
mir-4725	SF00988	mir-4787	SF00408	mir-4917	SF00469
mir-4726	SF01963	mir-4788	SF01178	mir-4918	SF02565
mir-4727	SF02015	mir-4789	SF00530	mir-4919	SF00750
mir-4728	SF01702	mir-4790	SF01783	mir-492	SF01210
mir-4729	SF00878	mir-4791	SF00657	mir-4920	SF00887
mir-4730	SF01180	mir-4792	SF00581	mir-4921	SF00810
mir-4731	SF01440	mir-4793	SF00801	mir-4922	SF01316
mir-4732	SF01396	mir-4794	SF01635	mir-4923a	SF01345
mir-4733	SF01542	mir-4795	SF00591	mir-4923b	SF01346
mir-4734	SF01492	mir-4796	SF02272	mir-4924	SF02326
mir-4735	SF01986	mir-4797	SF01506	mir-4925	SF02444
mir-4736	SF00442	mir-4798	SF00981	mir-4926	SF00888
mir-4737	SF02659	mir-4799	SF01884	mir-4927	SF02159
mir-4738	SF01256	mir-48	SF01117	mir-4929	SF02998
mir-4739	SF02920	mir-4800	SF00678	mir-493	SF00004
mir-4740	SF00798	mir-4801	SF02111	mir-4930	SF00992
mir-4741	SF01891	mir-4802	SF02075	mir-4931	SF02763
mir-4742	SF01672	mir-4803	SF00824	mir-4932	SF02809
mir-4743	SF01586	mir-4804	SF02684	mir-4933	SF02087
mir-4744	SF02710	mir-4805	SF02988	mir-4934	SF02226
mir-4745	SF02109	mir-4806	SF02847	mir-4935	SF02963
mir-4746	SF01703	mir-4807	SF00257	mir-4936	SF02445
mir-4747	SF01000	mir-4808	SF02995	mir-4937	SF01563
mir-4748	SF02749	mir-4809	SF02324	mir-4938	SF01843
mir-4749	SF00592	mir-4810	SF00256	mir-4939	SF02895
mir-4750	SF01174	mir-4811	SF02855	mir-493a	SF00615
mir-4751	SF00850	mir-4812	SF01498	mir-493b	SF00004
mir-4752	SF00789	mir-4813	SF00777	mir-494	SF00031
mir-4753	SF01073	mir-4814	SF02898	mir-4940	SF01478

miRNA Names	miR Family	miRNA Names	miR Family	miRNA Names	miR Family
mir-4941	SF02167	mir-503	SF00438	mir-515	SF00003
mir-4942	SF02281	mir-504	SF00317	mir-516	SF00003
mir-4943	SF02731	mir-5046	SF02457	mir-516a	SF00003
mir-4944	SF02391	mir-5047	SF00641	mir-516b	SF00003
mir-4945	SF01608	mir-505	SF00470	mir-517	SF00003
mir-4946	SF02648	mir-506	SF00099	mir-517a	SF00003
mir-4947	SF01959	mir-507	SF00099	mir-517b	SF00003
mir-4948	SF00385	mir-508	SF00099	mir-517c	SF00003
mir-4949	SF01083	mir-509	SF00099	mir-518a	SF00003
mir-495	SF00031	mir-5095	SF00001	mir-518b	SF00003
mir-4950	SF02962	mir-5096	SF00001	mir-518c	SF00003
mir-4951	SF01577	mir-5097	SF00969	mir-518d	SF00003
mir-4952	SF01400	mir-5098	SF00913	mir-518e	SF00003
mir-4953	SF01792	mir-5099	SF02056	mir-518f	SF00003
mir-4954	SF02934	mir-509a	SF00099	mir-519a	SF00003
mir-4955	SF02791	mir-509b	SF00099	mir-519b	SF00003
mir-4956	SF00455	mir-51	SF00666	mir-519c	SF00003
mir-4957	SF03015	mir-510	SF00099	mir-519d	SF00003
mir-4958	SF02577	mir-5100	SF01405	mir-519e	SF00003
mir-4959	SF02200	mir-5101	SF00751	mir-519f	SF00003
mir-496	SF00031	mir-5102	SF02447	mir-52	SF00667
mir-4960	SF01857	mir-5103	SF02406	mir-520a	SF00003
mir-4961	SF02704	mir-5104	SF01937	mir-520b	SF00003
mir-4962	SF02046	mir-5105	SF02792	mir-520c	SF00003
mir-4963	SF01795	mir-5106	SF00867	mir-520d	SF00003
mir-4964	SF00424	mir-5107	SF03004	mir-520e	SF00003
mir-4965	SF02342	mir-5108	SF02047	mir-520f	SF00003
mir-4966	SF02606	mir-5109	SF01676	mir-520g	SF00003
mir-4967	SF01168	mir-511	SF00504	mir-520h	SF00003
mir-4968	SF00234	mir-5110	SF01663	mir-521	SF00003
mir-4969	SF00142	mir-5111	SF01531	mir-522	SF00003
mir-497	SF01152	mir-5112	SF00511	mir-523	SF00003
mir-4970	SF00573	mir-5113	SF02118	mir-523a	SF00003
mir-4971	SF00143	mir-5114	SF01064	mir-523b	SF00003
mir-4972	SF02356	mir-5115	SF00560	mir-524	SF00003
mir-4973	SF00002	mir-5116	SF01053	mir-525	SF00003
mir-4974	SF02251	mir-5117	SF02966	mir-526a	SF00003
mir-4975	SF00987	mir-5118	SF00961	mir-526b	SF00003
mir-4976	SF01582	mir-5119	SF00201	mir-527	SF00003
mir-4977	SF01280	mir-512	SF00003	mir-53	SF00667
mir-4978	SF01953	mir-5120	SF02645	mir-532	SF00082
mir-4979	SF01047	mir-5121	SF02644	mir-539	SF00031
mir-498	SF01221	mir-5122	SF02467	mir-54	SF01163
mir-4980	SF01499	mir-5123	SF02596	mir-540	SF02550
mir-4981	SF00284	mir-5124	SF00134	mir-541	SF00341
mir-4982	SF00783	mir-5125	SF02698	mir-542	SF00309
mir-4983	SF02431	mir-5126	SF01620	mir-543	SF00031
mir-4984	SF01887	mir-5127	SF00260	mir-544	SF00001
mir-4985	SF01913	mir-5128	SF01483	mir-544a	SF00001
mir-4986	SF02008	mir-5129	SF02738	mir-544b	SF00001
mir-4987	SF02411	mir-5130	SF02591	mir-545	SF00350
mir-499	SF00603	mir-5131	SF02671	mir-546	SF00706
mir-499a	SF02786	mir-5132	SF01173	mir-547	SF00099
mir-5	SF00130	mir-5133	SF02794	mir-548a	SF00030
mir-50	SF01441	mir-5134	SF01043	mir-548aa	SF00368
mir-500	SF00082	mir-5135	SF02269	mir-548ab	SF00030
mir-500a	SF00082	mir-5136	SF02374	mir-548ac	SF00030
mir-500b	SF00082	mir-513a	SF00099	mir-548ad	SF00030
mir-501	SF00082	mir-513b	SF00099	mir-548ae	SF00030
mir-502	SF00082	mir-513c	SF00099	mir-548ag	SF00030
mir-502a	SF00082	mir-514	SF00099	mir-548ah	SF00030
mir-502b	SF00082	mir-514b	SF00099	mir-548ai	SF00030

miRNA Names	miR Family	miRNA Names	miR Family	miRNA Names	miR Family
mir-548aj	SF00030	mir-572	SF01971	mir-626	SF00047
mir-548ak	SF00030	mir-573	SF00792	mir-627	SF01904
mir-548al	SF00030	mir-574	SF00367	mir-628	SF00372
mir-548am	SF00030	mir-575	SF01946	mir-628a	SF00372
mir-548an	SF00030	mir-576	SF00723	mir-629	SF01181
mir-548b	SF00030	mir-577	SF00631	mir-63	SF01154
mir-548c	SF00030	mir-578	SF00773	mir-630	SF02651
mir-548d	SF00030	mir-579	SF00674	mir-631	SF00214
mir-548e	SF00030	mir-58	SF00854	mir-632	SF00176
mir-548f	SF00030	mir-580	SF01362	mir-633	SF02700
mir-548g	SF00030	mir-581	SF00979	mir-634	SF00334
mir-548h	SF00030	mir-582	SF00325	mir-635	SF00508
mir-548i	SF00030	mir-583	SF02321	mir-636	SF01282
mir-548j	SF00030	mir-584	SF00471	mir-637	SF00584
mir-548k	SF00030	mir-585	SF02561	mir-638	SF00739
mir-548l	SF00030	mir-586	SF00632	mir-639	SF00451
mir-548m	SF00030	mir-587	SF02071	mir-63a	SF00866
mir-548n	SF00030	mir-588	SF02253	mir-63b	SF00454
mir-548o	SF00030	mir-589	SF01538	mir-63c	SF00454
mir-548p	SF00030	mir-58a	SF00854	mir-63d	SF00349
mir-548q	SF00030	mir-58b	SF00854	mir-63e	SF00349
mir-548s	SF00821	mir-59	SF02500	mir-63f	SF00454
mir-548t	SF00030	mir-590	SF01848	mir-63g	SF00349
mir-548u	SF00030	mir-591	SF00400	mir-63h	SF00349
mir-548v	SF00030	mir-592	SF00440	mir-64	SF01130
mir-548w	SF00030	mir-593	SF00858	mir-640	SF01187
mir-548x	SF00030	mir-595	SF01079	mir-641	SF02332
mir-548y	SF00030	mir-596	SF01888	mir-642	SF01320
mir-549	SF02840	mir-597	SF00984	mir-642b	SF01320
mir-54a	SF01163	mir-598	SF02181	mir-643	SF00802
mir-54b	SF01163	mir-599	SF00249	mir-644	SF01901
mir-54c	SF01163	mir-6	SF00019	mir-645	SF02619
mir-54d	SF01163	mir-60	SF01644	mir-646	SF00407
mir-55	SF01268	mir-600	SF02516	mir-647	SF00721
mir-550	SF00339	mir-601	SF01602	mir-648	SF00525
mir-550a	SF00339	mir-602	SF01866	mir-649	SF01049
mir-550b	SF00554	mir-603	SF00030	mir-64a	SF01909
mir-551	SF00412	mir-604	SF00366	mir-64b	SF00295
mir-551a	SF00412	mir-605	SF00652	mir-64c	SF00294
mir-551b	SF00412	mir-606	SF02319	mir-64d	SF00474
mir-552	SF02471	mir-607	SF01545	mir-64e	SF00473
mir-553	SF02308	mir-608	SF01666	mir-65	SF01130
mir-554	SF01207	mir-609	SF02021	mir-650	SF00417
mir-555	SF01503	mir-61	SF02197	mir-650a	SF00417
mir-556	SF01285	mir-610	SF02195	mir-650b	SF00417
mir-557	SF00648	mir-611	SF01667	mir-650c	SF00417
mir-558	SF01518	mir-612	SF02070	mir-650d	SF00417
mir-559	SF02480	mir-613	SF00610	mir-651	SF02163
mir-55a	SF01855	mir-614	SF00393	mir-652	SF00099
mir-55b	SF02144	mir-615	SF00021	mir-653	SF00794
mir-56	SF01504	mir-616	SF00361	mir-654	SF00820
mir-561	SF00054	mir-617	SF02848	mir-655	SF00031
mir-562	SF00296	mir-618	SF02147	mir-656	SF00031
mir-563	SF00679	mir-619	SF00001	mir-657	SF02009
mir-564	SF00274	mir-61a	SF02113	mir-658	SF00818
mir-566	SF00022	mir-62	SF00958	mir-659	SF01305
mir-567	SF01385	mir-620	SF01998	mir-66	SF01393
mir-568	SF00291	mir-621	SF00593	mir-660	SF00082
mir-569	SF02533	mir-622	SF01675	mir-661	SF01469
mir-57	SF01625	mir-623	SF01929	mir-662	SF00323
mir-570	SF00030	mir-624	SF01376	mir-663	SF00912
mir-571	SF00808	mir-625	SF02657	mir-663a	SF00912

miRNA Names	miR Family	miRNA Names	miR Family	miRNA Names	miR Family
mir-663b	SF00912	mir-707	SF02581	mir-769	SF00111
mir-664	SF00567	mir-708	SF00006	mir-769b	SF00111
mir-664b	SF00567	mir-709	SF01463	mir-77	SF02050
mir-665	SF01614	mir-71	SF00171	mir-770	SF00092
mir-666	SF02726	mir-710	SF01871	mir-78	SF01287
mir-667	SF02654	mir-711	SF00767	mir-784	SF01420
mir-668	SF00235	mir-713	SF00115	mir-785	SF01228
mir-669	SF00347	mir-717	SF02583	mir-786	SF00758
mir-669a	SF00022	mir-718	SF01657	mir-787	SF02384
mir-669b	SF00022	mir-719	SF02387	mir-788	SF01072
mir-669c	SF00022	mir-71b	SF00770	mir-789	SF01560
mir-669d	SF00022	mir-72	SF00719	mir-79	SF00267
mir-669e	SF00022	mir-720	SF00044	mir-790	SF00212
mir-669f	SF00022	mir-721	SF01809	mir-791	SF01023
mir-669g	SF00022	mir-722	SF01118	mir-792	SF02469
mir-669h	SF00022	mir-723	SF01140	mir-793	SF01961
mir-669i	SF00022	mir-724	SF00314	mir-794	SF01732
mir-669j	SF00022	mir-725	SF02084	mir-795	SF02647
mir-669k	SF00022	mir-726	SF00345	mir-796	SF02177
mir-669l	SF00022	mir-727	SF02041	mir-797	SF02787
mir-669m	SF00022	mir-728	SF00972	mir-798	SF01813
mir-669n	SF00376	mir-729	SF01063	mir-799	SF00949
mir-669o	SF00022	mir-73	SF01490	mir-7a	SF00534
mir-669p	SF00022	mir-730	SF00623	mir-7b	SF00534
mir-67	SF01566	mir-731	SF00726	mir-8	SF00226
mir-670	SF00976	mir-732	SF02695	mir-80	SF00950
mir-671	SF00118	mir-733	SF02220	mir-800	SF00805
mir-672	SF00035	mir-734	SF02000	mir-802	SF00238
mir-673	SF02638	mir-735	SF01270	mir-804	SF02456
mir-674	SF02233	mir-736	SF00342	mir-81	SF00493
mir-675	SF01323	mir-737	SF01529	mir-82	SF00493
mir-675a	SF01323	mir-738	SF01145	mir-83	SF00076
mir-675b	SF01323	mir-739	SF00659	mir-84	SF00441
mir-676	SF00157	mir-73b	SF01490	mir-84a	SF01949
mir-677	SF02525	mir-74	SF00627	mir-84b	SF01949
mir-678	SF02707	mir-740	SF02602	mir-85	SF02338
mir-679	SF02343	mir-741	SF01826	mir-86	SF00233
mir-680	SF02621	mir-742	SF00099	mir-87	SF00381
mir-681	SF01394	mir-743a	SF00099	mir-871	SF00099
mir-682	SF00608	mir-743b	SF00099	mir-872	SF01100
mir-683	SF00697	mir-744	SF01902	mir-873	SF00566
mir-684	SF00957	mir-745	SF01249	mir-874	SF01164
mir-686	SF01724	mir-74a	SF00507	mir-875	SF01086
mir-687	SF01999	mir-74b	SF00507	mir-876	SF00154
mir-688	SF01603	mir-75	SF02231	mir-877	SF01055
mir-690	SF01090	mir-750	SF02094	mir-878	SF00099
mir-691	SF01205	mir-751	SF00769	mir-879	SF02509
mir-692	SF01487	mir-752	SF01019	mir-87a	SF01364
mir-693	SF02956	mir-753	SF02213	mir-87b	SF01363
mir-694	SF00555	mir-754d	SF00617	mir-880	SF00099
mir-695	SF02679	mir-757	SF02867	mir-881	SF00099
mir-697	SF02632	mir-758	SF00031	mir-882	SF01113
mir-698	SF00681	mir-759	SF01372	mir-883	SF00099
mir-7	SF00534	mir-76	SF00192	mir-883a	SF00099
mir-70	SF02140	mir-760	SF00790	mir-883b	SF00099
mir-700	SF02394	mir-761	SF01229	mir-885	SF02038
mir-701	SF01335	mir-762	SF00730	mir-887	SF00500
mir-702	SF02180	mir-763	SF00886	mir-888	SF00099
mir-703	SF00280	mir-764	SF00763	mir-889	SF00031
mir-704	SF01972	mir-765	SF00918	mir-890	SF00099
mir-705	SF00278	mir-766	SF00562	mir-891	SF01375
mir-706	SF01237	mir-767	SF00600	mir-891a	SF01375

miRNA Names	miR Family
mir-891b	SF01375
mir-892	SF00099
mir-892a	SF00099
mir-892b	SF00099
mir-9	SF00267
mir-90	SF01863
mir-90b	SF01863
mir-92	SF00009
mir-920	SF02028
mir-921	SF02735
mir-922	SF01276
mir-924	SF02626
mir-927	SF00461
mir-927b	SF02345
mir-928	SF01926
mir-929	SF00013
mir-92a	SF00009
mir-92b	SF00009
mir-92c	SF00009
mir-92d	SF00009
mir-92e	SF02605
mir-93	SF00038
mir-932	SF00302
mir-933	SF02790
mir-934	SF01182
mir-935	SF02351
mir-936	SF00868
mir-937	SF01658
mir-938	SF01847
mir-939	SF00575
mir-93a	SF00038
mir-93b	SF00038
mir-940	SF01637
mir-941	SF02508
mir-942	SF02939
mir-943	SF01765
mir-944	SF02100
mir-95	SF00350
mir-954	SF01886
mir-955	SF01729
mir-956	SF00265
mir-957	SF00524
mir-958	SF01414
mir-959	SF01002
mir-96	SF00747
mir-960	SF01548
mir-961	SF01465
mir-962	SF02625
mir-963	SF01192
mir-964	SF02089
mir-965	SF00276
mir-966	SF00065
mir-967	SF02756
mir-968	SF01755
mir-969	SF01177
mir-970	SF00258
mir-971	SF00845
mir-972	SF02184
mir-973	SF02559
mir-974	SF01535
mir-975	SF01355
mir-976	SF02984
mir-977	SF02138
mir-978	SF00682
mir-978a	SF00682
mir-978b	SF02354
mir-979	SF02359
mir-98	SF00057
mir-980	SF00702
mir-981	SF00046
mir-982	SF00484
mir-982a	SF00484
mir-982b	SF00484
mir-982c	SF00484
mir-983	SF02729
mir-984	SF01912
mir-985	SF00963
mir-986	SF01259
mir-987	SF00533
mir-988	SF00165
mir-989	SF02064
mir-99	SF00096
mir-990	SF01255
mir-991	SF01678
mir-992	SF02876
mir-993	SF00096
mir-993b	SF00096
mir-994	SF01085
mir-995	SF00605
mir-996	SF00676
mir-997	SF01526
mir-998	SF00785
mir-999	SF00694
mir-99a	SF00096
mir-99b	SF00096
mir-9a	SF00267
mir-9b	SF00267
mir-9c	SF00267
mir-iab-4	SF00290
mir-iab-8	SF00290
