# Supplementary material for: Large-scale analysis of microRNA evolution
Source: BMC Genomics. 2012 Jun 6;13:218. doi: 10.1186/1471-2164-13-218 (PMC3497579; doi:10.1186/1471-2164-13-218)
Supplement: Additional file 2 — Figure S2.Cluster Length per Species. As in Figure 3 but without normalisation. [file 1471-2164-13-218-S2.pdf]

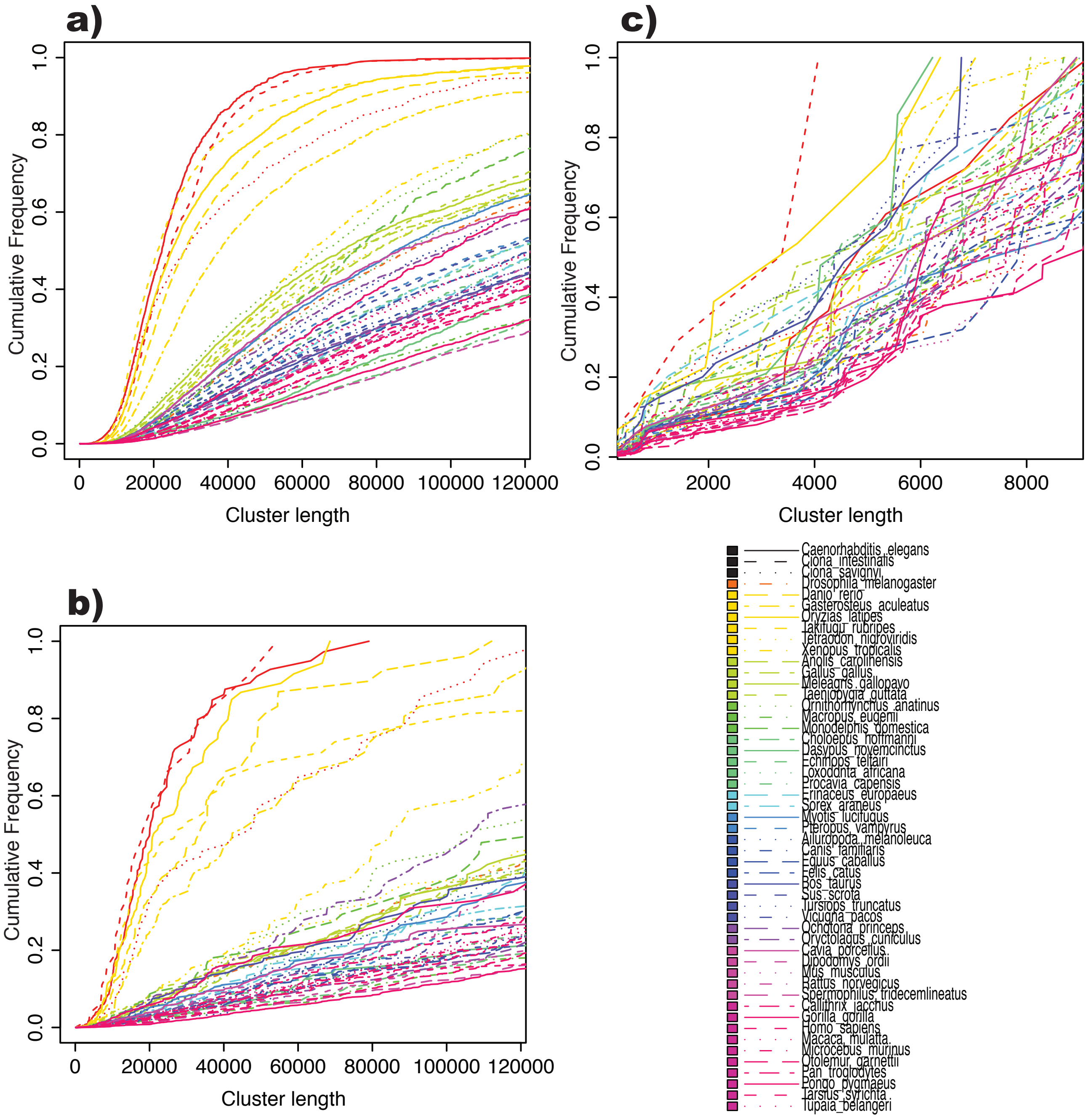
a)
b)
c)
Cumulative Frequency
Cluster length
0.0
0.2
0.4
0.6
0.8
1.0
0
20000
40000
60000
80000
100000
120000
2000
4000
6000
8000
Caenorhabditis_elegans
Ciona_intestinalis
Ciona_savignyi
Drosophila_melanogaster
Danio_rerio
Gasterosteus_aculeatus
Oryzias_latipes
Takifugu_rubripes
Tetraodon_nigroviridis
Xenopus_tropicalis
Anolis_carolinensis
Gallus_gallus
Meleagris_gallopavo
Taeniopygia_guttata
Ornithorhynchus_anatinus
Macropus_eugenii
Monodelphis_domestica
Choloepus_hoffmanni
Dasypus_novemcinctus
Echinops_telfairi
Loxodonta_africana
Procavia_capensis
Erinaceus_europaeus
Sorex_araneus
Myotis_lucifugus
Pteropus_vampyrus
Ailuropoda_melanoleuca
Canis_familiaris
Equus_caballus
Felis_catus
Bos_taurus
Sus_scrofa
Tursiops_truncatus
Vicugna_pacos
Ochotona_princeps
Oryctolagus_cuniculus
Cavia_porcellus
Dipodomys_ordii
Mus_musculus
Rattus_norvegicus
Spermophilus_tridecemlineatus
Callithrix_jacchus
Gorilla_gorilla
Homo_sapiens
Macaca_mulatta
Microcebus_murinus
Otolemur_garnettii
Pan_troglodytes
Pongo_pygmaeus
Tarsius_syrichta
Tupaia_belangeri
